# Aurora Kinase A Is Involved in Controlling the Localization of Aquaporin-2 in Renal Principal Cells

**DOI:** 10.3390/ijms23020763

**Published:** 2022-01-11

**Authors:** Sandrine Baltzer, Timur Bulatov, Christopher Schmied, Andreas Krämer, Benedict-Tilman Berger, Andreas Oder, Ryan Walker-Gray, Christin Kuschke, Kerstin Zühlke, Jenny Eichhorst, Martin Lehmann, Stefan Knapp, John Weston, Jens Peter von Kries, Roderich D. Süssmuth, Enno Klussmann

**Affiliations:** 1 Max-Delbrück-Center for Molecular Medicine in the Helmholtz Association (MDC), Robert-Rössle-Strasse 10, 13125 Berlin, Germany; sandrine.baltzer@mdc-berlin.de (S.B.); ryanwalkergray@gmail.com (R.W.-G.); christin.kuschke@mdc-berlin.de (C.K.); kerstin.zuehlke@mdc-berlin.de (K.Z.); 2 Institute of Chemistry, Technische Universität Berlin, Strasse des 17. Juni 135, 10623 Berlin, Germany; timur.bulatov@tu-berlin.de (T.B.); roderich.suessmuth@tu-berlin.de (R.D.S.); 3 Leibniz-Forschungsinstitut für Molekulare Pharmakologie (FMP), Robert-Rössle-Strasse 10, 13125 Berlin, Germany; schmied@fmp-berlin.de (C.S.); oder@fmp-berlin.de (A.O.); eichhorst@fmp-berlin.de (J.E.); mlehmann@fmp-berlin.de (M.L.); kries@fmp-berlin.de (J.P.v.K.); 4 Institute of Pharmaceutical Chemistry, Goethe University Frankfurt, Max-von-Laue-Strasse 9, 60438 Frankfurt am Main, Germany; kraemer@pharmchem.uni-frankfurt.de (A.K.); b.berger@chemie.uni-frankfurt.de (B.-T.B.); knapp@pharmchem.uni-frankfurt.de (S.K.); 5 Structural Genomics Consortium (SGC), Buchmann Institute for Molecular Life Sciences, Goethe University Frankfurt, Max-von-Laue-Strasse 15, 60438 Frankfurt am Main, Germany; 6 DKTK (German Translational Research Network), Partner Site Frankfurt/Mainz, 60590 Frankfurt am Main, Germany; 7 Frankfurt Cancer Institute, 60596 Frankfurt am Main, Germany; 8 JQuest Consulting, Carl-Orff-Weg 25, 65779 Kelkheim, Germany; westonjohn434@yahoo.co.uk; 9 DZHK (German Centre for Cardiovascular Research), Partner Site Berlin, 10785 Berlin, Germany

**Keywords:** AURKA, AQP2, AVP, cofilin-1, actin cytoskeleton

## Abstract

The cAMP-dependent aquaporin-2 (AQP2) redistribution from intracellular vesicles into the plasma membrane of renal collecting duct principal cells induces water reabsorption and fine-tunes body water homeostasis. However, the mechanisms controlling the localization of AQP2 are not understood in detail. Using immortalized mouse medullary collecting duct (MCD4) and primary rat inner medullary collecting duct (IMCD) cells as model systems, we here discovered a key regulatory role of Aurora kinase A (AURKA) in the control of AQP2. The AURKA-selective inhibitor Aurora-A inhibitor I and novel derivatives as well as a structurally different inhibitor, Alisertib, prevented the cAMP-induced redistribution of AQP2. Aurora-A inhibitor I led to a depolymerization of actin stress fibers, which serve as tracks for the translocation of AQP2-bearing vesicles to the plasma membrane. The phosphorylation of cofilin-1 (CFL1) inactivates the actin-depolymerizing function of CFL1. Aurora-A inhibitor I decreased the CFL1 phosphorylation, accounting for the removal of the actin stress fibers and the inhibition of the redistribution of AQP2. Surprisingly, Alisertib caused an increase in actin stress fibers and did not affect CFL1 phosphorylation, indicating that AURKA exerts its control over AQP2 through different mechanisms. An involvement of AURKA and CFL1 in the control of the localization of AQP2 was hitherto unknown.

## 1. Introduction

Arginine-vasopressin (AVP)-dependent water reabsorption in renal-collecting duct principal cells relies on the tight regulation of the cyclic adenosine monophosphate (cAMP) signaling pathway. The activation of vasopressin V2 receptors (V2R) on the basolateral plasma membrane of the principal cells by AVP induces an elevation of intracellular cAMP and the activation of cAMP-dependent protein kinase (protein kinase A, PKA) which, in turn, initiates signaling that eventually leads to the exocytic translocation of the water channel aquaporin-2 (AQP2) from intracellular vesicles into the plasma membrane of the cells [1,2]. This process is crucial for water reabsorption from primary urine. A dysregulated control of AQP2 causes or is associated with water balance disorders, such as diabetes insipidus [2,3].

The rearrangement of the actin cytoskeleton has emerged as a key regulator of AQP2 trafficking [3]. Filamentous (F-)actin has a dual role. It acts as a physical barrier preventing AQP2-bearing vesicles from gaining access to the plasma membrane, and thereby preventing inappropriate water reabsorption in the absence of AVP [4,5,6], or F-actin serves as tracks for AQP2-bearing vesicles en route to the plasma membrane [7,8]. Vesicle docking and fusion requires the transient removal of the dense cortical actin network, a process which is orchestrated by an interplay of F-actin and various actin regulatory proteins [9]. V2R stimulation induces the depolymerization of cortical F-actin and its accumulation at the lateral tight junctions [10]. In contrast to this actin-barrier model, actin filaments and their associated transport proteins can also act as a positive regulator of exocytosis by driving the transport of vesicles to the plasma membrane and their docking to the cell cortex [8,11]. During intracellular trafficking, AQP2 binds to actin both directly via a fragment in its C-terminus [12] or indirectly via interaction with a range of actin-binding proteins [8]. The actin cytoskeleton thereby not only provides anchor points for AQP2-bearing vesicles but also a network for their intracellular trafficking [13]. It was also suggested that actin filaments serve as tracks for the translocation of AQP2 from early endosomes to subapical storage compartments after its retrieval from the plasma membrane [14]. Immunoisolation of AQP2-bearing vesicles from rat inner medullary collecting ducts and their analysis by proteomics revealed the presence of actin cytoskeletal proteins, Rab GTPases, SNARE, and myosin motor proteins, all of which may contribute to the trafficking process [15]. For instance, tropomyosin-5b (TM5b) interacts with AQP2 and the actin cytoskeleton [12,16]. This interaction was highly dynamic and regulated by cAMP signaling and AQP2 phosphorylation at serine (Ser) 256 by PKA [16].

Aurora kinase A (AURKA) belongs to a family of highly conserved serine/threonine protein kinases of which three mammalian paralogues, AURKA, AURKB, and AURKC, exist [17,18]. The paralogues differ in sequence length but share a common domain organization consisting of an N-terminal domain that contains the adenosine triphosphate (ATP)-binding site, a central catalytic domain, and a short C-terminal domain. The catalytic domain is highly conserved with 71%, 60%, and 75% sequence homology between AURKA/B, AURKA/C, and AURKB/C, respectively [18,19]. Both AURKA and AURKB are key regulators of mitotic cell division and are expressed in all tissues; AURKC is restricted to meiosis in germ cells. The paralogues vary in their (sub)cellular localization and exhibit different substrate affinities and binding partners. Their expression is cell cycle-dependent [17,18,19]. The intracellular function of AURKA and AURKB is to a large extent determined by their location, which is not only defined by their conserved C-terminus but also their divergent N-terminal kinase domains [20,21,22]. AURKA and AURKB have been extensively studied in the context of cancer as their genes are frequently overexpressed/amplified in solid tumors and hematological malignancies [23,24,25,26]. This and the fact that they regulate and interact with a variety of substrates that are involved in oncogenic pathways has made them an attractive target for cancer therapy and prompted the development of selective ATP-competitive kinase inhibitors [27]. Various AURK inhibitors have been developed over the past years, some of which are used as tools in molecular biology (e.g., Aurora-A inhibitor I [28]) or undergo clinical trials (e.g., Alisertib [29] or Barasertib-HQPA and its prodrug Barasertib [30]). The AURKA-selective inhibitor Alisertib has passed phase III, but none of the kinase inhibitors have yet been approved as an anti-cancer drug [29]. Recently, selective AURKA PROTACs (protein-targeting chimeras) have also been developed [31,32].

AURKA phosphorylates the actin regulatory protein cofilin-1 (CFL1) and thereby affects actin remodeling during mitosis [33]. We had previously carried out an siRNA screening in immortalized mouse medullary collecting duct (MCD4) cells to identify kinase-related genes involved in the control of AQP2. The screening identified 19 kinases whose knockdown inhibited the cAMP-induced redistribution of AQP2, amongst them AURKA [34]. We therefore further investigated a potential role of AURKA in the control of AQP2. We report here an involvement of AURKA and CFL1 in the control of the localization of AQP2 in two renal principal cell models. Within this context we synthesized new AURKA-selective small molecule inhibitors, which provide valuable insights into the structure–activity relationship of the Aurora-A inhibitor I. To our knowledge, a connection between CFL1 as actin regulator and AQP2 trafficking or a role of AURKA in this process is hitherto unknown.

## 2. Results

### 2.1. Inhibition of AURKA Prevents the cAMP-Induced Redistribution of AQP2 

**Figure 1:**
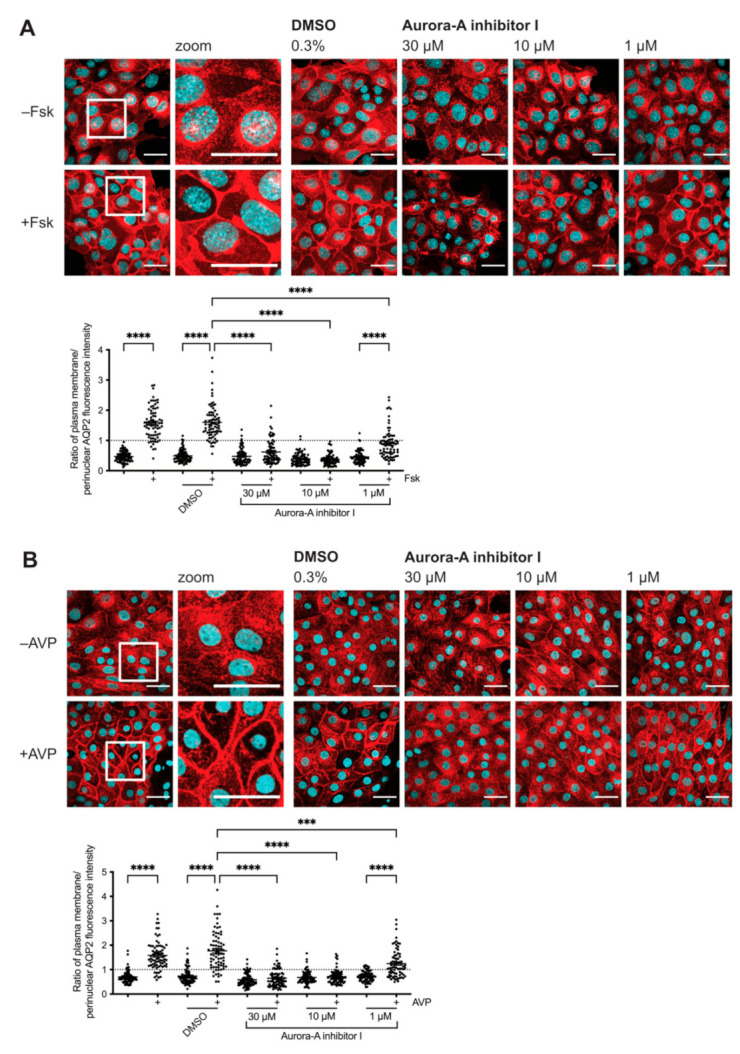
Aurora-A inhibitor I inhibits the cyclic adenosine monophosphate (cAMP)-induced aquaporin-2 (AQP2) redistribution to the plasma membrane of (**A**) immortalized mouse medullary collecting duct (MCD4) and (**B**) primary rat inner medullary collecting duct (IMCD) cells. The cells were left untreated or treated with DMSO (0.3%, 1 h) or Aurora-A inhibitor I (1 µM, 10 µM or 30 µM, 1 h) alone or in combination with forskolin (Fsk; 30 µM, 30 min) or arginine-vasopressin (AVP; 100 nM, 30 min) where indicated. AQP2 was detected by immunofluorescence microscopy using specific primary antibody (AQP2, E-2) and Cy3-coupled anti-mouse secondary antibody (red). Nuclei were stained with DAPI (cyan). Shown are representative images of three independent experiments; scale bar 30 µm. Magnified views (zoom) are from the indicated boxes. The intensities of plasma membrane and perinuclear immunofluorescence signals arising from AQP2 were determined. The ratios of plasma membrane to perinuclear fluorescence signal intensities were calculated. Ratios >1 indicate a predominant localization at the plasma membrane. Shown are means ± SEM of three independent experiments with a total of 75 cells per condition. Statistically significant differences compared to DMSO-treated control cells are indicated, **** *p* < 0.0001, *** *p* < 0.001.

In our previous siRNA screening we had identified AURKA as a potential regulator of the AQP2 redistribution downstream of PKA [34]. To validate the inhibitory effect of the knockdown of AURKA on the AQP2 redistribution, we examined the effect of a selective AURKA inhibitor (Aurora-A inhibitor I; TC-S 7010) on the AQP2 localization in our two cell models for AQP2 trafficking, MCD4 and primary rat inner medullary collecting duct (IMCD) cells (Figure 1). Aurora-A inhibitor I is a potent inhibitor of AURKA with an IC_50_ of 3.4 nM in a cell-free assay (Table 1; [28]). It is 1000-fold more selective for AURKA over AURKB. Aurora-A inhibitor I induces G2/M cell cycle arrest, multinucleation, and apoptosis in ARID1A-deficient colorectal cancer cells [35].

MCD4 and primary IMCD cells were treated with Aurora-A inhibitor I prior to their stimulation. Unlike primary IMCD cells, which can be stimulated with AVP, MCD4 cells lack the V2R. Their stimulation is achieved by the cAMP-elevating agent forskolin, which directly activates adenylyl cyclases. In resting cells, AQP2 is mainly localized on intracellular vesicles in the perinuclear area. In response to forskolin or AVP, AQP2 is redistributed to the plasma membrane. The AQP2 localization was analyzed by immunofluorescence microscopy (Figure 1). The AQP2 fluorescence signals at the plasma membrane and perinuclear area of the cells were determined, and the ratios of plasma membrane to perinuclear AQP2 fluorescence intensities were calculated. A ratio >1 indicated a predominant localization at the plasma membrane, while ratios <1 indicated a predominant intracellular localization.

The AURKA-selective inhibitor was effective in concentrations greater than 1 µM and inhibited the redistribution in both cell models. We did not observe the inhibitory effect in all cells most likely because the expression of AURKA is cell cycle-dependent [18], and unsynchronized MCD4 cells might be differentially affected by AURKA inhibition depending on their cell cycle state, density, and general proliferation rate. 

The use of compound concentrations in the low µM range is common for the principal models in this study. Other examples include the antimycotic drug fluconazole [36], inhibitors of the interaction of AKAP-Lbc with RhoA [37], or Rho inhibitors [4]. The apparently high concentrations are most likely due to the robust plasma membranes of the cells, which, in situ in the kidney are exposed to various kinds of adverse environmental factors, including nephrotoxic agents in serum or urine. In the further experiments in primary IMCD cells, compounds were applied in the low µM concentration range, which did not alter the cell shape.

### 2.2. Inhibition of AURKA but Not AURKB Prevents the cAMP-Induced Redistribution of AQP2 

**Figure 2:**
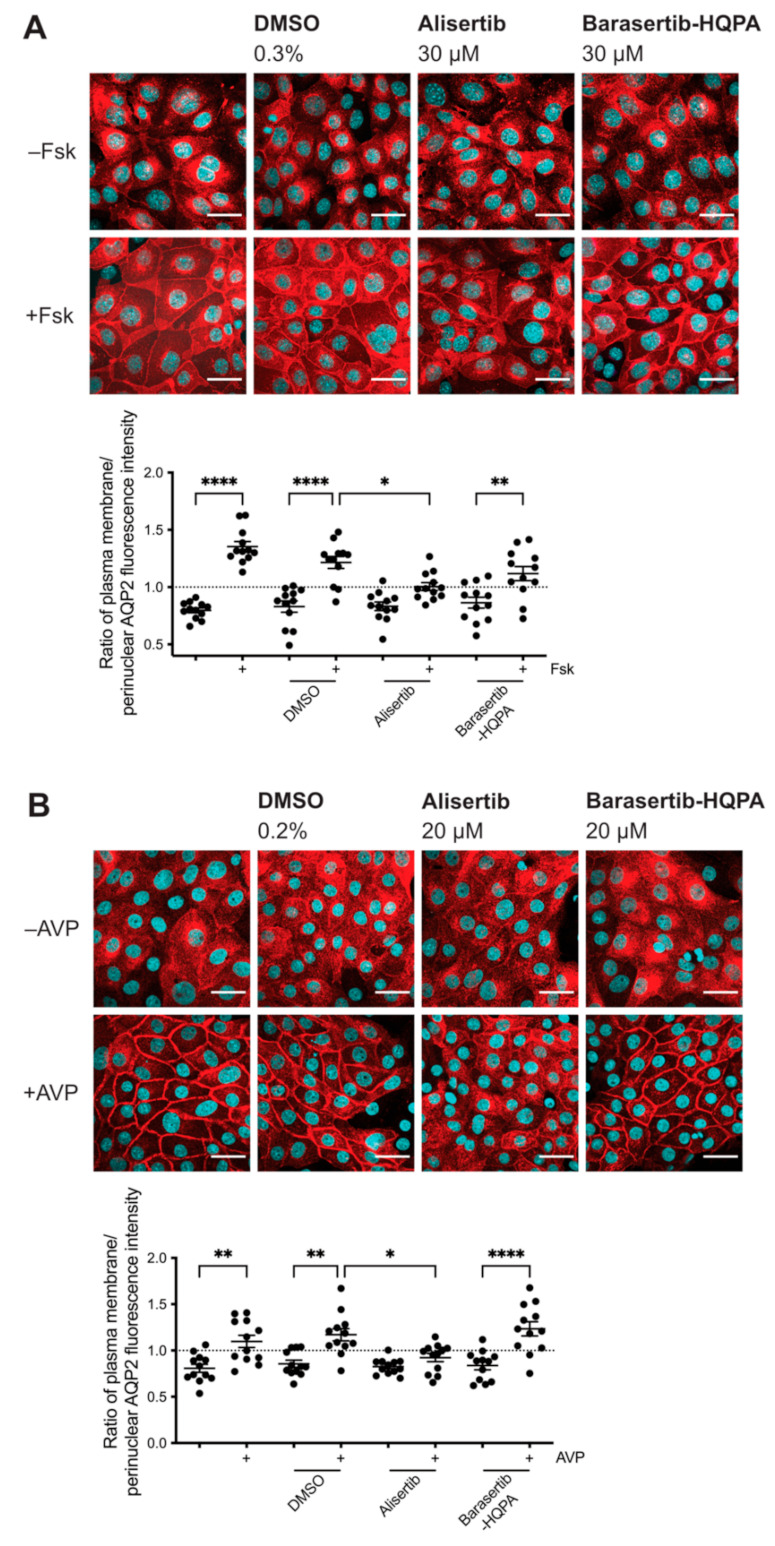
Inhibition of Aurora kinase A (AURKA) but not Aurora kinase B (AURKB) inhibits the redistribution of AQP2. (**A**) MCD4 or (**B**) primary IMCD cells were left untreated or treated with DMSO (0.3% or 0.2%, 1 h), Alisertib (AURKA inhibitor; 30 µM or 20 µM, 1 h), or Barasertib-HQPA (AURKB inhibitor; 30 µM or 20 µM, 1 h) alone or in combination with forskolin (Fsk; 30 µM, 30 min) or arginine-vasopressin (AVP; 100 nM, 30 min) where indicated. AQP2 was detected by immunofluorescence microscopy as described in the legend of Figure 1. Shown are representative images of four independent experiments; scale bar 30 µm. AQP2 was quantified using a segmentation-based approach [42]. Ratios > 1 indicate a predominant localization at the plasma membrane. Shown are means ± SEM of four independent experiments with ≥25 cells per image per condition. Statistically significant differences are indicated, **** *p* < 0.0001, ** *p* < 0.01, * *p* < 0.05.

Next, we sought to define whether the observed inhibitory effect on the AQP2 redistribution was due to inhibition of AURKA and not one of its paralogues. We thus treated our cells with another commercially available AURKA-selective inhibitor, Alisertib (MLN8237), as well as an AURKB-selective inhibitor, Barasertib-HQPA (AZD-1152-HQPA; Figure 2, Table 1). Alisertib has an IC_50_ of 1.2 nM in a cell-free assay and >200-fold higher selectivity for AURKA over AURKB [38]. Aurora-A inhibitor I has antiproliferative effects [38,39]. Barasertib-HQPA is a potent AURKB inhibitor with an IC_50_ of 0.37 nM in a cell-free assay and a ~3800-fold selectivity for AURKB compared to AURKA [40,41]. We excluded AURKC-selective inhibitors from testing as the expression of AURKC has been mainly reported for germ cells [18]. We thus expected that the biological effects we observed in our renal cell models could only be elicited by the activity of AURKA or AURKB.

We treated MCD4 cells with the inhibitors and analyzed the AQP2 localization by immunofluorescence microscopy (Figure 2A). Alisertib significantly inhibited the forskolin-induced redistribution of AQP2, whereas Barasertib-HQPA did not. Intrigued by the AURKA-specific effect, we continued by testing the effect of the inhibitors in our primary cell model. In primary IMCD cells, AURKA inhibition by Alisertib significantly inhibited the redistribution compared to control cells treated with the compound solvent, DMSO. No inhibitory effect on the AQP2 redistribution was visible after inhibiting AURKB (Figure 2B). The observed inhibitory effects on the AQP2 redistribution elicited by two structurally different AURKA inhibitors (Table 1), Aurora-A inhibitor I and Alisertib, demonstrated an AURKA-specific role in the control of the AQP2 localization.

### 2.3. Aurora-A Inhibitor I Derivatives Show Varying Effects on the AQP2 Localization

**Figure 3:**
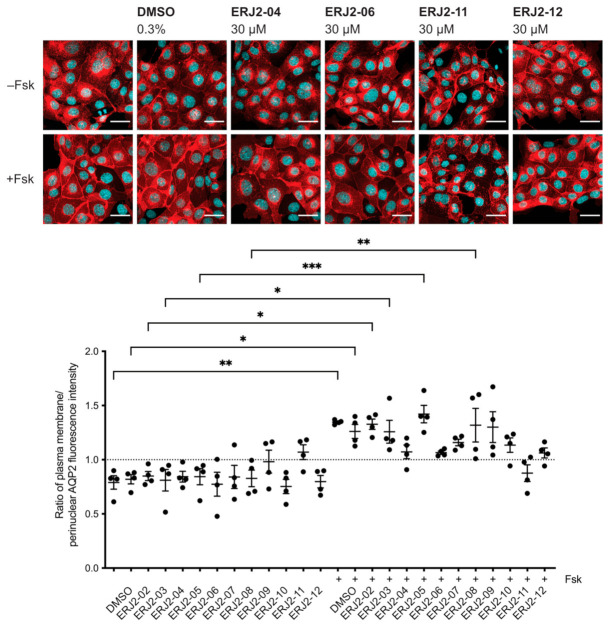
Aurora-A inhibitor I derivatives inhibit the forskolin-induced redistribution of AQP2 to the plasma membrane of MCD4 cells. Cells were left untreated or treated with DMSO (0.3%, 1 h) or compound (30 µM, 1 h) alone or in combination with forskolin (Fsk; 30 µM, 30 min). AQP2 was detected by immunofluorescence microscopy as described in the legend of Figure 1. Shown are representative images of four independent experiments; scale bar 30 µm. AQP2 was quantified using a segmentation-based approach [42]. Ratios > 1 indicate a predominant localization at the plasma membrane. Shown are means ± SEM of four independent experiments with ≥25 cells per image per condition. Statistically significant differences between resting and forskolin-stimulated cells are indicated, *** *p* < 0.001, ** *p* < 0.01, * *p* < 0.05. Additional images can be found in Appendix A (Figure A1).

**Figure 4:**
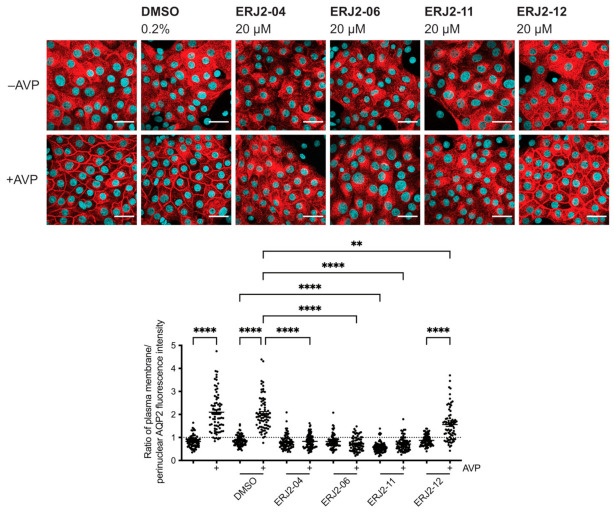
Aurora-A inhibitor I derivatives inhibit the AVP-induced redistribution of AQP2 to the plasma membrane of primary IMCD cells. Cells were left untreated or treated with DMSO (0.2%, 1 h) or compound (20 µM, 1 h) alone or in combination with arginine-vasopressin (AVP; 100 nM, 30 min). AQP2 was detected by immunofluorescence microscopy and quantified as described in the legend of Figure 1. Shown are representative images of three independent experiments; scale bar 30 µm. Ratios > 1 indicate a predominant localization at the plasma membrane. Shown are means ± SEM of three independent experiments with a total of 75 cells per condition. Statistically significant differences compared to DMSO-treated control cells are indicated, **** *p* < 0.0001, ** *p* < 0.01.

We aimed to further elucidate the relationship between the chemical structure of Aurora-A inhibitor I and its biological activity as an inhibitor of AURKA and the AQP2 redistribution. Preparation of Aurora-A inhibitor I derivatives by medicinal chemistry [28] yielded a new set of compounds (Table 2). We analyzed their effects on the AQP2 localization by immunofluorescence microscopy. In MCD4 cells, the derivatives showed varying inhibitory effects on the forskolin response (Figure 3). In the presence of forskolin in combination with either ERJ2-04, -06, -11, or -12, the localization of AQP2 was not different from that in cells treated with the compound solvent, DMSO. Therefore, being amongst the most effective inhibitors of the AQP2 redistribution, ERJ2-04, -06, -11, and -12 were chosen for further testing in primary IMCD cells (Figure 4). There was no significant difference between resting and AVP-stimulated cells that had been treated with ERJ2-04, -06, or -11 but AQP2 fluorescence intensity ratios significantly differed for ERJ2-12-treated cells. A significant decrease in the AQP2 fluorescence intensity ratio compared to DMSO-treated control cells was detected upon treatment with ERJ2-11. We occasionally observed prominent AQP2 speckles in the perinuclear area upon treatment with this compound (not shown), which might explain the shift towards lower AQP2 intensity ratios.

### 2.4. Cell Viability Is Affected by Some of the Aurora-A Inhibitor I Derivatives

**Figure 5:**
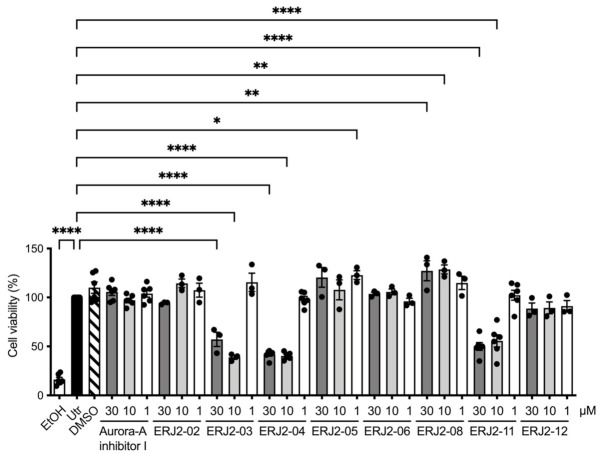
Effects of Aurora-A inhibitor I and its derivatives on the viability of MCD4 cells. Cell proliferation was assessed using the CellTiter 96^®^ Aq_ueous_ One Solution Cell Proliferation Assay (Promega; Walldorf, Germany). MCD4 cells were left untreated (Utr) or treated with DMSO (0.3%, 1 h) or compound (30 µM, 10 µM or 1 µM, 1 h). As negative controls, i.e., to induce maximal toxicity, cells were incubated with ethanol (EtOH). Shown are means ± SEM of three or six independent experiments. Statistically significant differences compared to untreated cells are indicated, **** *p* < 0.0001, ** *p* < 0.01, * *p* < 0.05.

Antiproliferative effects elicited by Aurora-A inhibitor I had previously been described for the colon cancer cell lines HCT116 and, to a smaller extent, HT-29 [28,35]. None of our inhibitors induced apparent cell shape changes in MCD4 or primary IMCD cells. However, in order to investigate the effects on the viability of our test cells upon inhibition of AURKA, we carried out MTS assays (Figure 5). MTS assays rely on the conversion of a tetrazolium compound into a colored formazan product by NADPH or NADH in metabolically active cells [43,44]. They are based on the assumption that the production is proportional to the number of viable cells. 

Amongst the AURKA inhibitors with inhibitory properties on the AQP2 redistribution, Aurora-A inhibitor I and ERJ2-06 did not elicit any significant effects on the viability of MCD4 cells within the 1 h of treatment. Cell viability was reduced by 10 or 30 µM of ERJ2-03, -04, or -11. ERJ2-03 and -04 both have a morpholine moiety and ERJ2-11 a piperazine moiety. As morpholine and piperazine are well-known drug-like features, these moieties are unlikely to cause the reduction in cell viability, suggesting that another part of these compounds exerts the deleterious effect on MCD4 cell viability [45,46]. This is supported by the observation that ERJ2-12, which also has a piperazine fragment, does not affect the viability of MCD4 cells. With ERJ2-03 not being an inhibitor of the AQP2 redistribution (Figure 3), reduced viability of the MCD4 cells did not necessarily seem to be accompanied by an impaired AQP2 trafficking. Incubation with ERJ2-05 or -08 partly increased the viability compared to untreated control cells. This could be due to interference of the compound with the colorimetric assay itself or could be due to off-target effects in the cells. The observation that the Aurora-A inhibitor I did not affect viability of MCD4 cells was reassuring that AURKA is directly involved in the control of AQP2.

### 2.5. Aurora-A Inhibitor I and Its Derivatives Show Favorable Selectivity Profiles

**Figure 6:**
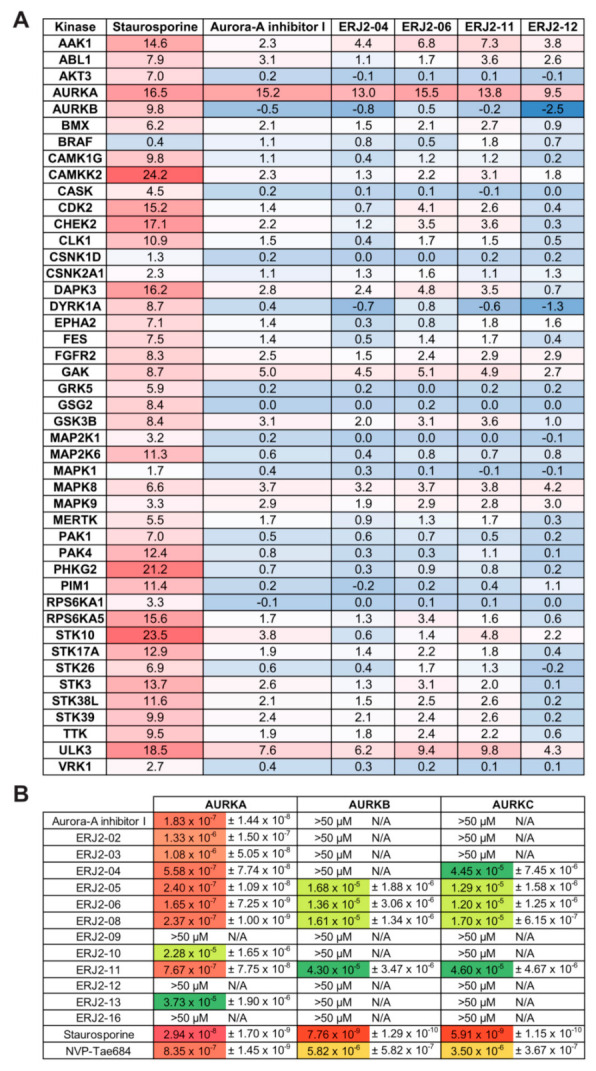
Aurora-A inhibitor I and its derivatives are mostly selective for AURKA. (**A**) Thermal shift assay to determine binding of Aurora-A inhibitor I and its derivatives to different kinases. Staurosporine served as a positive control; *n* = 3. Unit ΔT_m_ (°C). (**B**) The affinity of the test compounds to intracellular AURKA/B/C was determined by NanoBRET measurements. Staurosporine and NVP-Tae684 served as positive controls; *n* = 2; shown are means ± SD. Affinities are listed in molar concentration (M) if not stated otherwise. Affinities are color-coded with the lowest values on dark red background; lowest binding is indicated by no background.

Next, we confirmed the specificity of our new Aurora-A inhibitor I derivatives by evaluating binding to 45 different kinases in a thermal shift assay [47] (Figure 6A). The binding of a compound to a kinase domain induces a measurable shift in its melting temperature (ΔTm). ΔTm values are unique for each kinase, i.e., binding affinities cannot be directly derived, and ΔTm values have to be interpreted in the context of a positive control such as staurosporine, a global kinase inhibitor. Our measurements confirmed a strong affinity of the tested compounds to AURKA, underpinned by ΔTm values in the range of the positive control. Aurora-A inhibitor I and ERJ2-06 induced the highest ΔTm. None of the compounds bound to AURKB. Moderate ΔTm values were measured for a small number of other serine/threonine kinases such as AP2-associated protein kinase 1 (AAK1), Cyclin G-associated kinase (GAK), and Unc-51like kinase 3 (ULK3). However, those shifts were not in the range of the positive control. To our knowledge, a role of these kinases in the control of AQP2 has not been investigated.

In quantitative phosphoproteomic analyses, AAK1 was shown to be phosphorylated upon V2R activation in renal collecting duct cells [48,49]. However, its inhibition by our AURKA inhibitors is unlikely because inhibition of clathrin-mediated endocytosis in renal principal cells causes an accumulation of AQP2 in the plasma membrane [50]. In a transcriptional profiling study, only GAK and the ULK3 homologue ULK1 were found to be expressed in rat IMCD cells [51]. GAK also regulates clathrin-mediated endocytosis [52,53], and its inhibition by AURKA inhibitors as a reason for the inhibition of the AQP2 redistribution is unlikely for the same reason as for AAK1: GAK inhibition would be expected to cause an accumulation of AQP2 in the plasma membrane [50]. In addition, GAK also contributes to centrosome maturation and mitotic progression [54]. Due to its high plasticity, the kinase domain of GAK is known to be predestined to off-targeting by different clinical kinase inhibitors [55] and thus potentially also by our AURKA inhibitors. Conformational plasticity of the kinase domain has also been reported for ULK3 [56]. ULK3 is involved in Sonic Hedgehog (SHH) signaling [57,58] and the induction of autophagy as a consequence of cellular senescence [59]. 

The selectivity of the Aurora-A inhibitor I derivatives against the three mammalian AURK paralogues was determined by a NanoBRET assay, by which we measured the apparent affinities of our compounds to the respective kinases in HEK293T cells overexpressing the individual kinases (Figure 6B). Most compounds bound AURKA with affinities in the nanomolar range and displayed higher selectivity for AURKA over the two AURK paralogues. The most potent inhibitor of the AQP2 redistribution, ERJ2-06, showed the strongest affinity for AURKA binding (165 nM). Surprisingly, ERJ2-12 did not bind to any of the AURK paralogues, despite inducing a moderate temperature stabilization in the thermal shift assay (Figure 6A). This might be due to limited cell penetration of this inhibitor and provides a rationale for the ambiguous results observed for MCD4 and primary IMCD cells (Figure 3 and Figure 4). Moreover, a high cellular ATP concentration acts as a competitive binder to the kinase domains.

On the structural level, the ortho-chlorophenyl on the right-hand side of Aurora-A inhibitor I and ERJ2-02 to -08 appeared to be very important for the compound’s activity as an AURKA inhibitor, possibly as it allows a conformationally preferred conformation. Derivatives lacking this group were poor or weaker binders of AURKA (Table 2, Figure 6B). Substitution of the ortho-chlorophenyl by an unsubstituted phenyl rendered the molecules less active (ERJ2-10 and -11). However, ERJ2-11 still potently inhibited the forskolin response in MCD4 cells (Figure 3). Derivatives lacking the ortho-chlorophenyl or unsubstituted phenyl, e.g., ERJ2-12, were inactive. Substitutions of the terminal fragment at the left-hand side were of little importance, and most variations were tolerated. This was in line with previously published work on ortho-chlorophenyl substituted pyrimidines [60].

**Figure 7:**
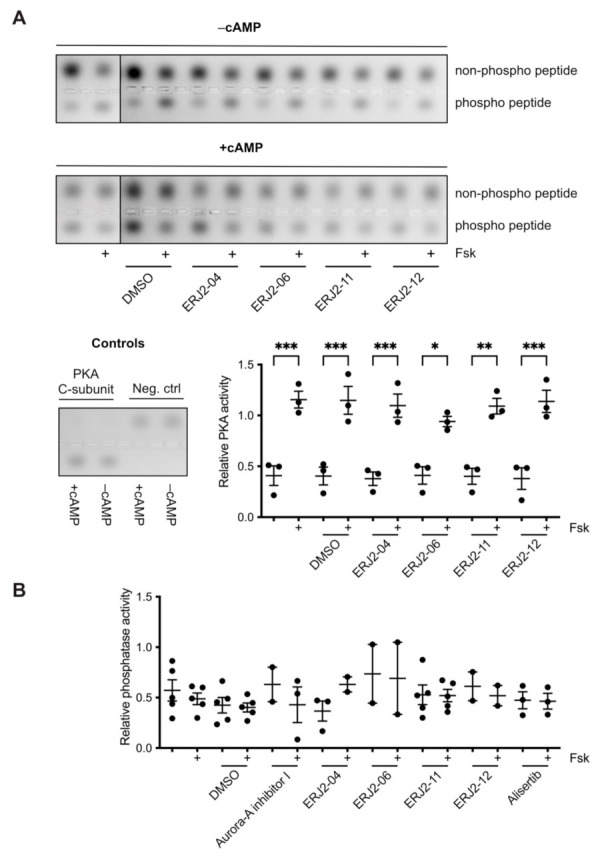
AURKA inhibitors do not affect global protein kinase A (PKA) or protein phosphatase activity in MCD4 cell lysates. (**A**) PKA activity in MCD4 cell lysates was determined using the PepTag^®^ Non-Radioactive Protein Kinase Assay (Promega; Walldorf, Germany). Cells were left untreated or treated with DMSO (0.3%, 1 h) or compound (30 µM, 1 h) alone or in combination with forskolin (Fsk; 30 µM, 30 min). cAMP (1 µM) was added to induce maximal PKA activity. PKA catalytic (C) subunit (2 µg/mL) was used in positive controls and omitted in negative controls. Relative PKA activity was determined by calculating the ratio of phosphorylated to non-phosphorylated peptides, which were semi-quantitatively analyzed by densitometry. Shown are representative agarose gels and means ± SEM of three independent experiments. Statistically significant differences are indicated, *** *p* < 0.001, ** *p* < 0.01, * *p* < 0.05. (**B**) Phosphatase activity in MCD4 cell lysates was determined by para-Nitrophenylphosphate (pNPP)-based activity assays. Cells were left untreated or treated with DMSO (0.3%, 1 h) or compound (30 µM, 1 h) alone or in combination with forskolin (Fsk; 30 µM, 30 min). Cell lysates were incubated with a pNPP substrate solution and absorbance measured at 405 nm. Shown are means ± SEM of two to five independent experiments.

We next carried out PKA activity assays to rule out off-target effects of the compounds on PKA (Figure 7A). None of the tested compounds significantly reduced the forskolin response in MCD4 cell lysates compared to forskolin-stimulated control lysates. Global effects of the derivatives on phosphatase activity in MCD4 cells were assessed by a para-Nitrophenylphosphate (pNPP)-based activity assays. No significant differences compared to control lysates were detected (Figure 7B).

### 2.6. The Inhibition of AURKA Affects the Organization of the Actin Cytoskeleton in Both MCD4 and Primary IMCD Cells

Actin remodeling is orchestrated by a variety of actin regulatory proteins including AURKA [33]. We therefore investigated whether the observed inhibitory effect on the AQP2 redistribution by AURKA inhibition could be explained by its role in the regulation of the actin cytoskeleton. For this, we stained F-actin in our cells using a fluorophore-coupled phalloidin to investigate the inhibitors’ effects on the actin cytoskeleton by immunofluorescence microscopy. 

**Figure 8:**
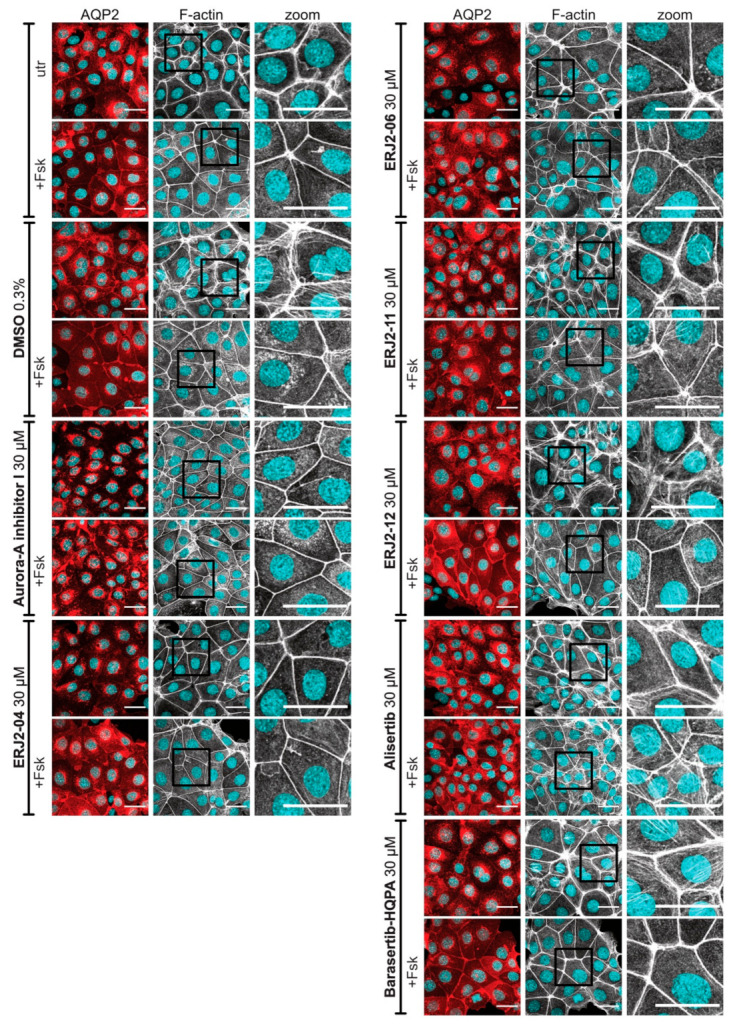
AURKA inhibitors affect the organization of actin stress fibers in MCD4 cells. MCD4 cells were left untreated (utr) or treated with DMSO (0.3%, 1 h) or compound (30 µM, 1 h) alone or in combination with forskolin (Fsk; 30 µM, 30 min). AQP2 was detected by immunofluorescence microscopy using specific primary antibody (AQP2, E-2) and Cy3-coupled anti-mouse secondary antibody (red). Filamentous (F-)actin was stained using an iFluor 488-conjugated phalloidin (grey). Nuclei were stained with DAPI (cyan). Shown are representative images of three independent experiments; scale bar 30 µm. Magnified views (zoom) of actin stress fibers are from the indicated boxes.

**Figure 9:**
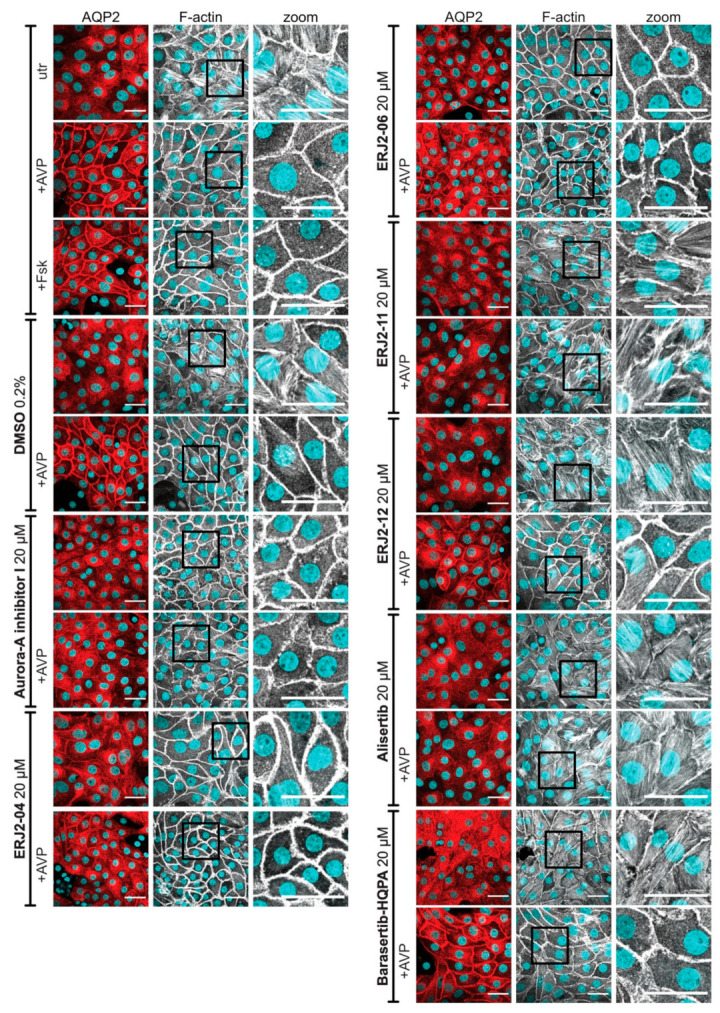
AURKA inhibitors affect the organization of actin stress fibers in primary IMCD cells. Primary IMCD cells were left untreated (utr) or treated with DMSO (0.2%, 1 h) or compound (20 µM, 1 h) alone or in combination with forskolin (Fsk; 30 µM, 30 min) or arginine-vasopressin (AVP; 100 nM, 30 min). AQP2 was detected by immunofluorescence microscopy using specific primary antibody (AQP2, E-2) and Cy3-coupled anti-mouse secondary antibody (red). F-actin was stained using an iFluor 488-conjugated phalloidin (grey). Nuclei were stained with DAPI (cyan). Shown are representative images of three independent experiments; scale bar 30 µm. Magnified views (zoom) of actin stress fibers are from the indicated boxes.

Resting principal cells exhibit a network of F-actin-containing stress fibers, which depolymerizes upon cAMP elevation [4]. Accordingly, forskolin and AVP decreased the actin stress fibers in both MCD4 (Figure 8) and primary IMCD cells (Figure 9). Treatment with Aurora-A inhibitor I led to the depolymerization of actin stress fibers even in the absence of a cAMP-elevating agent. Comparable effects were observed for ERJ2-04 and ERJ2-06. Surprisingly, the other AURKA inhibitor, Alisertib, and also ERJ2-11 promoted or maintained the formation of actin stress fibers. The phenotypes of cells treated with ERJ2-12 and Barasertib-HQPA resembled those of the control cells. We concluded that the AURKA inhibitors may act via different mechanisms on the AQP2 localization.

### 2.7. The Inhibition of AURKA by Aurora-A Inhibitor I Decreases the Phosphorylation of the AURKA Substrate CFL1 in Both MCD4 and Primary IMCD Cells

CFL1 is a substrate of AURKA and is involved in the control of the actin cytoskeleton [33]. In its active unphosphorylated form, CFL1 depolymerizes F-actin. We hypothesized that the inhibition of AURKA by our inhibitors might lead to a reduction in phosphorylated (p-)CFL1. This would cause a depolymerization of the actin cytoskeleton even in the absence of forskolin/AVP as observed for Aurora-A inhibitor I, ERJ2-04 and -06 (Figure 8 and Figure 9). However, it would not account for the effects observed after treatment with ERJ2-11 and Alisertib.

**Figure 10:**
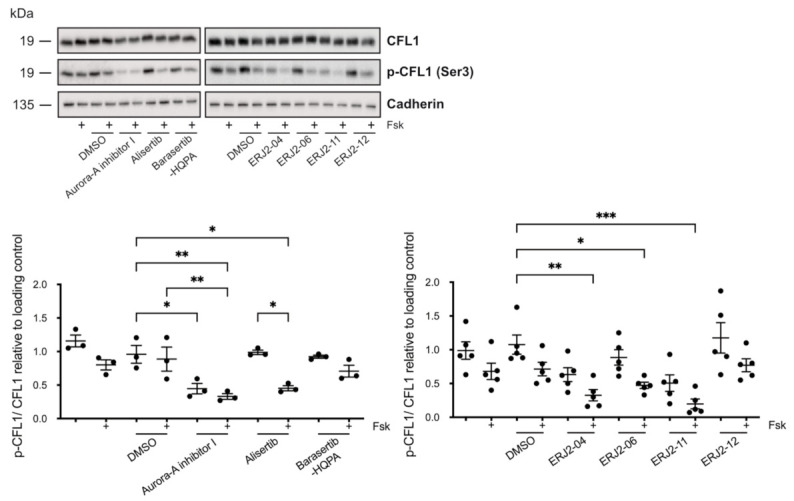
AURKA inhibitors affect the phosphorylation of cofilin-1 (CFL1) in MCD4 cells. Cells were left untreated or treated with DMSO (0.3%, 1 h) or compound (30 µM, 1 h) alone or in combination with forskolin (Fsk; 30 µM, 30 min). Cell lysates were prepared and CFL1, phosphorylated (p-)CFL1, and cadherin as loading control were detected by Western blotting using specific antibodies. The signals were semi-quantitatively analyzed by densitometry and normalized to the loading control. Shown are representative blots and means ± SEM of three to five independent experiments. Statistically significant differences compared to DMSO-treated control cells are indicated, *** *p* < 0.001, ** *p* < 0.01, * *p* < 0.05.

We prepared lysates of MCD4 cells that had been incubated with the AURK inhibitors and analyzed the samples by Western blotting (Figure 10). Indeed, in accordance with its actin stress fiber-depolymerizing effect, Aurora-A inhibitor I significantly decreased the CFL1 phosphorylation both in the absence and presence of forskolin. Significant additive effects in combination with forskolin were observed for Aurora-A inhibitor I, ERJ2-04, -06, and -11. AURKA inhibition by the compounds most likely induced a priming effect on the CFL1 dephosphorylation, which is enforced by forskolin. Alisertib elicited a similar effect in forskolin-stimulated cells. However, in contrast to Aurora-A inhibitor I-treated cells, CFL1 phosphorylation was not reduced in resting cells. Compounds that did not inhibit AURKA (ERJ2-12 and Barasertib-HQPA) had no effect on the CFL1 phosphorylation.

**Figure 11:**
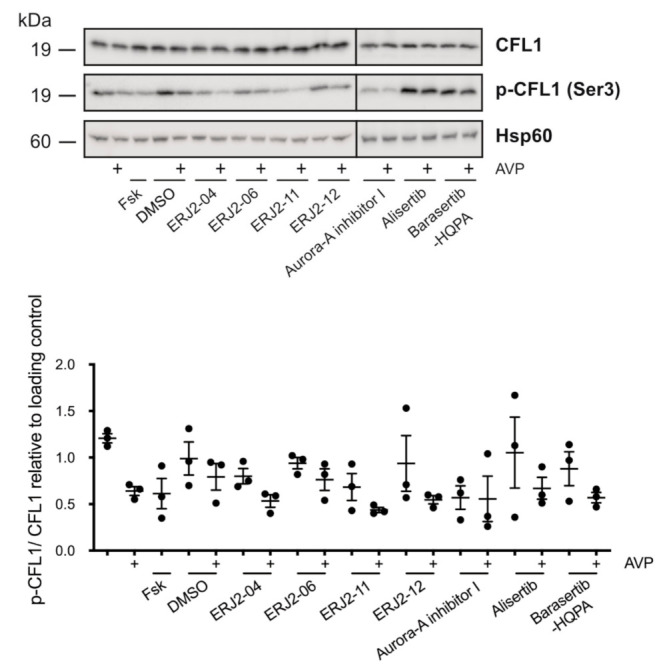
AURKA inhibitors affect the phosphorylation of CFL1 in primary IMCD cells. Cells were left untreated or treated with DMSO (0.2%, 1 h) or compound (20 µM, 1 h) alone or in combination with forskolin (Fsk; 30 µM, 30 min) or arginine-vasopressin (AVP; 100 nM, 30 min). Cell lysates were prepared and CFL1, phosphorylated (p-)CFL1 and Hsp60 as loading control were detected by Western blotting using specific antibodies. The signals were semi-quantitatively analyzed by densitometry and normalized to the loading control. Shown are representative blots and means ± SEM of three independent experiments.

In primary IMCD cell lysates, no significant changes in the CFL1 phosphorylation could be detected (Figure 11). However, densitometric analysis of the Western blot samples revealed trends comparable to the results obtained in MCD4 cells (Figure 10). Relatively low levels of CFL1 phosphorylation were detected after incubation of the cells with Aurora-A inhibitor I or one of its active derivatives. 

Taken together, the effects on the CFL1 phosphorylation upon treatment of MCD4 and primary IMCD cells with Aurora-A inhibitor I and its structurally closely related derivatives ERJ2-04 and -06 were in line with their depolymerizing effect on the actin cytoskeleton (Figure 8 and Figure 9). An exception is ERJ2-11, which showed a tendency to promote or maintain the formation of actin stress fibers. This could be explained by structural differences as ERJ2-11 lacks the ortho-chlorophenyl as described before. The two selective AURKA inhibitors Aurora-A inhibitor I and Alisertib differ in their chemical structure, which most likely explains their different effects on the actin cytoskeleton and CFL1 phosphorylation. Collectively, our data show that AURKA exerts the control over the AQP2 localization through different mechanisms: It modulates the actin cytoskeleton through CFL1 phosphorylation (Aurora-A inhibitor I) and through modulating another target and thereby another signaling pathway (Alisertib). 

### 2.8. The Inhibition of AURKA and AURKB Has no Effect on the Phosphorylation of LIM Kinase-1 (LIMK-1) in Primary IMCD Cells

**Figure 12:**
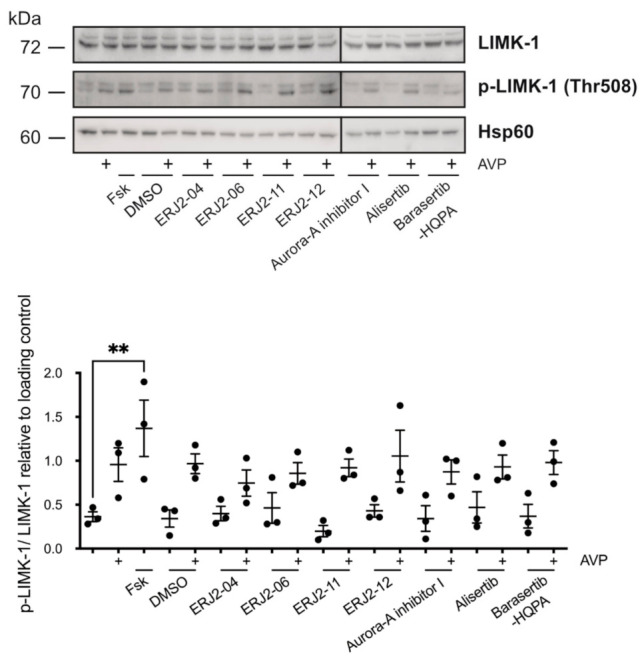
AURKA and AURKB inhibitors do not affect the phosphorylation of LIM kinase-1 (LIMK-1) in primary IMCD cells. Cells were left untreated or treated with forskolin (Fsk; 30 µM, 30 min), DMSO (0.2%, 1 h), or compound (20 µM, 1 h) alone or in combination with arginine-vasopressin (AVP; 100 nM, 30 min). Cell lysates were prepared and LIMK-1, phosphorylated (p-)LIMK-1 (threonine (Thr) 508), and Hsp60 as loading control were detected by Western blotting using specific antibodies. The signals were semi-quantitatively analyzed by densitometry and normalized to the loading controls. Shown are representative blots and means ± SEM of three independent experiments. Statistically significant differences are indicated, ** *p* < 0.01.

Besides AURKA, LIMK-1 as well as phosphatases such as slingshot phosphatase 1 (SSH-1) and chronophin (CIN) regulate CFL1 activity by phosphorylation or dephosphorylation, respectively [33,61,62,63,64]. In primary IMCD cell lysates we could not detect significant changes in the phosphorylation of LIMK-1 upon treatment of the cells with the compounds (Figure 12). In contrast to AVP, forskolin induced a significant increase in the LIMK-1 phosphorylation. This can most likely be explained by the compartmentalization of cAMP signaling: Forskolin was previously shown to induce higher cAMP levels and relative PKA activity compared to AVP in primary IMCD cells. This was due to global activation of PKA, in contrast to focal activation induced by AVP [65]. On the level of phosphatases, we could not detect global effects of the compounds on phosphatase activity (Figure 7B).

## 3. Discussion

**Figure 13:**
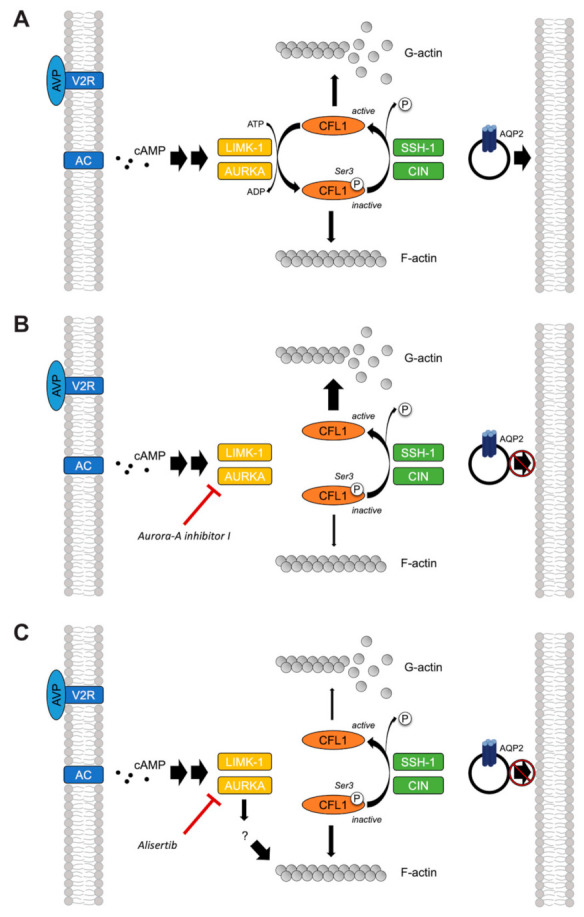
Aurora-A inhibitor I and Alisertib inhibit the redistribution of aquaporin-2 (AQP2) in renal principal cells by different mechanisms. (**A**) Under physiological conditions, the binding of arginine-vasopressin (AVP) to the vasopressin V2 receptor (V2R) activates adenylyl cyclase (AC) and increases intracellular cyclic adenosine monophosphate (cAMP). The redistribution of AQP2 to the plasma membrane involves actin remodeling. The actin regulatory protein cofilin-1 (CFL1) is phosphorylated (P) at serine (Ser) 3 and thereby inactivated by LIM kinase 1 (LIMK-1) and Aurora kinase A (AURKA). Inactive CFL1 promotes the formation and maintenance of filamentous (F-)actin stress fibers. The depolymerization of F-actin into globular (G-)actin monomers is induced after reactivation of CFL1 by dephosphorylation by slingshot phosphatase 1 (SSH-1) and chronophin (CIN). (**B**) Aurora-A inhibitor I inhibits AURKA and decreases the CFL1 phosphorylation in stimulated cells. It thereby promotes the F-actin depolymerization. (**C**) Alisertib inhibits AURKA and increases the amount of F-actin stress fibers without affecting the CFL1 phosphorylation, indicating that another signaling pathway is involved.

In this study, we showed that two structurally different AURKA inhibitors, Aurora-A inhibitor I and Alisertib, inhibit the AQP2 redistribution in two renal principal cell models. Our findings demonstrate that AURKA is involved in the control of the AQP2 redistribution via at least two different pathways. We propose the mechanisms depicted in Figure 13.

AURKA inhibition by Aurora-A inhibitor I elicited a depolymerization of the actin cytoskeleton by reducing the AURKA-catalyzed phosphorylation of CFL1 (Figure 10), which increases its activity [33]. The observed depolymerization of F-actin-containing stress fibers was consistent in both our primary and permanent cell models (Figure 8 and Figure 9) and explains the inhibitory effect on the AQP2 redistribution, because actin stress fibers function as tracks that are necessary for the transport of AQP2-bearing vesicles to the plasma membrane [7,8]. 

The mode of action of Alisertib and its inhibitory effect on the redistribution of AQP2 cannot be explained by this mechanism. However, it apparently also involves the actin cytoskeleton because an increase in F-actin-containing stress fibers compared to control cells was observed (Figure 8 and Figure 9). F-actin has a dual role in the regulation of the AQP2 redistribution. It not only acts as tracks but in the cell periphery as cortical F-actin also as a physical barrier for AQP2-bearing vesicles, preventing them to reach the plasma membrane [4,5,6]. The partial depolymerization of F-actin, for example, by cytochalasin D, is sufficient to induce the redistribution of AQP2 to the plasma membrane, supporting the notion that cortical F-actin acts as a barrier for this translocation process [4,5]. Alisertib apparently strengthens this physical barrier.

The small GTPase RhoA promotes the formation of cortical F-actin and thereby inhibits the redistribution of AQP2. PKA inhibits RhoA activity, causing the depolymerization of cortical F-actin. This enables AQP2-bearing vesicles to reach the plasma membrane [4,5,66,67]. AURKA phosphorylates Drok, the Rho-kinase orthologue in *Drosophila*, in vivo and thereby inactivates it [68]. Further, the vertebrate Rho-kinase isoform p160ROCK is phosphorylated by AURKA [69]. Thus, the control over the AQP2 localization by AURKA could involve RhoA and/or its downstream effector ROCK. 

Downstream of RhoA/ROCK, actin remodeling is orchestrated by a complex interplay of LIMK-1 and AURKA [33,63,64,70], as well as phosphatases such as SSH-1 and CIN [61,62], which all regulate the activity of CFL1. A functional interaction has been reported for LIMK-1 and AURKA. During mitotic spindle formation, both kinases reciprocally serve as one other’s substrate and activate each other by phosphorylation [71]. We could not detect significant effects on the phosphorylation of LIMK-1 upon AURKA inhibition (Figure 12). LIMK-1 phosphorylates and thereby inactivates CFL1 [63,64]. A similar role has been suggested for AURKA. The kinase directly phosphorylates CFL1 at multiple sites, including at Ser3, and thereby regulates the organization of the actin cytoskeleton in early mitotic stages [33]. In this context, AURKA elicits an inhibitory effect on the CFL1 activity by blocking its binding to actin filaments. Opposite effects have been reported for cell migration and metastasis of various cancers such as papillary thyroid [72] and breast cancer [70], where overexpressed AURKA induced an up-regulation of SSH-1 expression, thus favoring the dephosphorylation and activation of CFL1. In M12 cells, AURKA inhibition by Alisertib increased the phosphorylation of CFL1, possibly as a consequence of decreased SSH-1 levels [33]. We could not detect an increased CFL1 phosphorylation upon treatment with Alisertib by Western blotting (Figure 10 and Figure 11). However, the results from this previous study could provide an explanation for the pronounced actin stress fibers detected in both resting and stimulated Alisertib-treated cells (Figure 8 and Figure 9) 

Taken together, the involvement of a target other than CFL1 in the control of the AQP2 redistribution seems likely because Alisertib did not elicit the same effects on the actin cytoskeleton and the CFL1 phosphorylation as Aurora-A inhibitor I despite also inhibiting the AQP2 redistribution. Belonging to different classes of compounds, the structural differences of Aurora-A inhibitor I and Alisertib most likely explain their distinct effects on the AURKA kinase activity and the affected downstream signaling pathway.

AURKA kinase activity is spatially and temporally regulated by complex mechanisms [17,29] and determined by the phosphorylation of the threonine (Thr) 288 residue in its activation loop, both by autophosphorylation or phosphorylation by other kinases [73,74]. Thr288 is part of a PKA consensus motif, and AURKA has been shown to become phosphorylated and thereby activated by PKA in vitro [74]. Further, AURKA is activated by mitogen- and stress-activated protein kinase (MSK1) in vitro [73]. We could not detect the phosphorylation of AURKA at Thr288 in our two cell models, possibly because its abundance was below the detection limit. Problems with the detection of p-AURKA signals have been previously reported and were explained by solubility issues, possibly by association of p-AURKA with insoluble cytoskeletal elements [75]. Moreover, most available AURKA and p-AURKA antibodies are reactive towards human AURKA and can thus not be used for the detection in mouse or rat cell lysates as it was the case in this study. Therefore, it is currently unclear whether V2R and subsequent PKA activation lead to the phosphorylation/activation of AURKA.

Different effects of Aurora-A inhibitor I and Alisertib in cells have been reported [75]. For instance, the two inhibitors differed in their affinity towards the complex of AURKA and its allosteric activator targeting protein for *Xenopus* kinesin-like protein 2 (TPX2). The affinity of Alisertib for AURKA was 5.8-fold decreased in the presence of TPX2, while the binding of Aurora-A inhibitor I to the complex was 2.4-fold increased [75]. These observations can be explained by the presence of different conformational states: The activation loop of the kinase is framed by conserved DFG and APE motifs (one-letter amino acid code) and can adopt different conformations. In the inactive DFG-out state, an additional hydrophobic binding site is exposed next to the ATP binding site [76,77]. Binding of Alisertib to AURKA promoted the inactive DFG-out state, which is counteracted by TPX2 binding to AURKA, which induced the active DFG-in state [78,79,80]. In contrast, binding of Aurora-A inhibitor I to the AURKA binding pocket is less affected as it has little contact with the structural elements, which move upon binding of TPX2 [28,75]. These conformational changes in the kinase induced by inhibitor binding might account for a conformational selectivity, determining the kinase’s substrates and which downstream signaling pathways it induces [78]. Such observations may explain why Aurora-A inhibitor I and Alisertib act via different pathways and why ERJ2-11, despite sharing structural similarity with Aurora-A inhibitor I, ERJ2-04 and -06, elicits different effects on the F-actin stress fibers. A group of ortho-chlorophenyl substituted pyrimidines, structurally resembling our ERJ compounds, was shown to bind in a DFG-out state [60]. ERJ2-11 lacks the ortho-chlorophenyl that might affect its DFG binding mode.

Based on the discovered different modes of action of Aurora-A inhibitor I and Alisertib with regard to inhibiting the AQP2 redistribution, we analyzed the effect of our compounds on other kinases using a thermal shift assay (Figure 7A). Profiling against a panel of kinases revealed possible interactions with the serine/threonine kinases AAK1, GAK, and ULK3. The role of these proteins in the control of the localization of AQP2 is unknown. They could potentially constitute additional components of the machinery controlling AQP2 in renal principal cells. Phosphoproteomic studies revealed an AVP/V2R-dependent signaling network in renal-collecting duct cells that involves the regulation of a plethora of kinases, partly in a PKA-independent manner [48,49,81]. We had previously identified 19 potential regulators of the AQP2 redistribution via siRNA-mediated knockdown of 719 kinase-related genes, one of which was AURKA [34]. However, a comprehensive understanding of the complete signaling pathways involving such a kinase network in the control of AQP2 in renal principal cells is lacking. 

A few examples indicate that AURKA does not only play a role in the kidney during mitosis. AURKA activates the vacuolar H^+^-ATPase (V-ATPase) in Caki-2 cells, a human kidney carcinoma cell line [82]. Inhibition of V-ATPase with 4-acetyldiphyllin (4AD) inhibited the cAMP-dependent AQP2 redistribution. An increase in the intravesicular pH and an accumulation of AQP2 within the Golgi compartment was induced by 4AD [83]. Thus, the inhibitory effect of AURKA may involve V-ATPase. Other non-mitotic functions have been reported for AURKA [84]. For instance, there is an interphase pool of AURKA in ciliated cells, regulating cilia disassembly [85], and neurons, contributing to neurite outgrowth [86]. Our finding that AURKA is involved in the control of AQP2 adds another example to the repertoire of AURKA’s non-mitotic functions and supports the notion that AURKA exerts physiological roles outside of the mitotic context. Moreover, the discovery of AURKA inhibitors for interfering with the AQP2 trafficking expands the toolbox of inhibitors that directly interfere with vasopressin-mediated water transport [87]. The discovery of novel inhibitors of the redistribution of AQP2 also strengthens arguments that modulating the localization of an aquaporin for therapeutic reasons is feasible. The inhibition of the translocation of AQP2 into the plasma membrane may be an approach to reduce edema in heart failure where elevated levels of AVP cause a predominant localization of AQP2 in the plasma membrane of the renal principal cells [2,88]. Recently, the translocation of AQP4 was prevented with the approved drug trifluoperazine [89]. The drug prevented the localization of AQP4 at the blood-spinal cord barrier and ablated CNS edema in a rat model. Of note, the cAMP-PKA pathway plays a critical role in the control of the localization of both AQP2 and AQP4, underpinning that the pathway is a viable therapeutic target despite being ubiquitous [88].

## 4. Materials and Methods

### 4.1. Chemical Synthesis of Novel Aurora-A Inhibitor I Derivatives

Instructions on the chemical synthesis of novel Aurora-A inhibitor I derivatives can be found in the Supplementary Materials (File S1).

### 4.2. Compounds

Commercial AURK inhibitors were purchased from Sigma-Aldrich, Taufkirchen, Germany (Aurora-A inhibitor I/TC-S 7010, #SML0882) and MedChemExpress, Köln, Germany (Alisertib/MLN8237, #HY10971; Barasertib-HQPA/AZD1152-HQPA, #HY-10126).

### 4.3. Cell Cultures

Primary rat IMCD cells were obtained and cultured as previously described [36,83,88,89]. MCD4 cells [34,36,90] were grown in DMEM/F-12 GlutaMAX^TM^ (Life Technologies, Darmstadt, Germany; #31331) supplemented with 5% (*v*/*v*) FCS and 5 µM of dexamethasone. MCD4 cells were used at 80–90% confluency. Their doubling time is ~26 h. The same batch of cells was used for the entire study. Cells were cultured up to passage 20–25. Changes in morphology and AQP2 expression were regularly monitored using an inverted microscope. Primary IMCD cells were seeded to be fully confluent (95–100%). Mycoplasma contamination of MCD4 cells was excluded prior to their freezing and storage in liquid nitrogen using commercially available kits.

### 4.4. Animal Treatment

The local authority (Landesamt für Gesundheit und Soziales, Berlin, Germany) and MDC had approved the use of animals for obtaining primary IMCD cells (Anzeige der Tötung von Wirbeltieren zu wissenschaftlichen Zwecken nach § 4 (3) TSchG). Rats (10–12 weeks, male, Wistar Han) were obtained from Charles River and kept on standard diet and tap water. 

### 4.5. Immunofluorescence Microscopy of MCD4 and Primary IMCD Cells

Cells were grown on 12 mm coverslips. Immunofluorescence staining was carried out as previously described [7,34,36,83,91] using the following primary and secondary antibodies (1:300): AQP2 (Santa Cruz Biotechnology, Heidelberg, Germany; E-2, sc-515770), ZO-1 (Santa Cruz Biotechnology, sc-33725), Cy3-F(ab’)_2_-anti-Mouse IgG (H+L, Jackson Immuno Research, Ely, UK; #115-166-003), Alexa647-anti-Rat IgG (H+L, Jackson Immuno Research, #712-605-150), and Cy2-anti-Rat IgG (H+L, Jackson Immuno Research, Ely, UK; #112-225-167). F-actin was visualized using Phalloidin-iFluor 488 Reagent (1:1000, Abcam, Cambridge, UK; ab176753). Nuclei were stained with 4′,6-Diamidino-2-phenylindol (DAPI, 1:100, Roche, Mannheim, Germany; #10236276001). 

Cells were imaged using a confocal laser scanning microscope (LSM) 710 (Carl Zeiss, Jena, Germany) with a Plan-Apochromat 63×/1.40 oil objective. Image analysis was performed using the software Fiji by Image J (Version 2.1.0) [92]. Where indicated, AQP2 localization was quantified using a segmentation-based approach as previously described [42]. For this, masks of the plasma membrane and perinuclear regions were generated and used for the determination of the fluorescence signal intensity arising from AQP2 in the respective cellular area.

Three to five fields of view were taken per sample and experiment. The sample’s identity was only revealed after taking representative images. Non-specific binding and background autofluorescence were assessed when establishing the staining protocol by omitting either the primary or secondary antibody and by an unlabeled sample. 

### 4.6. MTS Assays

The CellTiter 96^®^ Aqueous One Solution Cell Proliferation Assay (Promega, Walldorf, Germany) was used according to the manufacturer’s instructions and as previously described [37]. Cells were seeded in a 96-well format (30,000 cells/well) and grown over night. Cells were treated with compounds as indicated. As a control, ethanol (EtOH) was used to induce maximal toxicity. Wells containing medium only served as blanks. After 50 min, the MTS reagent was added to the cell suspension, and incubation at 37 °C continued for another 10 min. The absorbance at 490 nm was measured using a Spark^®^ 20M Multimode Reader.

### 4.7. Western Blotting

Cells were lysed in standard lysis buffer (SLB; 10 mM K_2_HPO_4_, 150 mM NaCl, 5 mM EDTA, 5 mM EGTA, 0.5% Triton X-100, 0.2% sodium deoxycholate; pH 7.4) supplemented with protease (cOmpleteTM, mini, EDTA-free; Roche, Mannheim, Germany; #1183617001) and phosphatase (PhosSTOP EASY pack; Roche, #04906837001) inhibitors, if not stated otherwise. 

Western blotting was carried out as previously described [34,36,37,83,93,94] using the following primary and secondary antibodies: Cofilin (1:2000, Cell Signaling, Frankfurt am Main, Germany; D3F9, #5175), p-Cofilin (Ser3, 1:1000, Cell Signaling, 77G2, #3313), Hsp60 (1:1000, Cell Signaling, D307, #4870), Pan-Cadherin (1:1000, Abcam, Cambridge, UK; ab6528), LIMK-1 (1:500, Santa Cruz Biotechnology, Heidelberg, Germany; H-84, sc-5576), p-LIMK-1/2 (Thr508/505, 1:200, Santa Cruz, sc-28409-R), POD-F(ab’)_2_-anti-Rabbit IgG (H+L, 1:5000, Jackson Immuno Research, Ely, UK; #711-036-152), and POD-anti-Mouse IgG (H+L, 1:5000, Jackson Immuno Research, #715-035-151). Signals were detected using Immobilon^®^ Western Chemiluminescent HRP Substrate (Merck, Darmstadt, Germany; #WBKLS0500) and an Odyssey^®^ Fc detection system (Li-Cor Biosciences, Bad Homburg vor der Höhe, Germany). Image processing and densitometric analysis was performed with the Li-Cor software Image Studio Lite (Ver. 5.2.5).

### 4.8. Differential Scanning Fluorimetry-Based Selectivity Screening against a Curated Kinase Library (Thermal Shift Assay)

The assay was performed as previously described [47,95]. Briefly, recombinant protein kinase domains (2 μM) were mixed with compound (10 µM) in 20 mM HEPES, pH 7.5 and 500 mM NaCl. SYPRO Orange (5000×, Invitrogen/Thermo Fisher Scientific, Dreieich, Germany) was added as a fluorescence probe (1 µL/mL). Temperature-dependent protein unfolding profiles were measured using the QuantStudio™ 5 real-time PCR machine (Thermo Fisher Scientific). Excitation and emission filters were set to 465 nm and 590 nm, respectively. The temperature was raised with a step rate of 3 °C per minute. Data points were analyzed with the internal software (Thermal Shift Software^TM^ Version 1.4, Thermo Fisher Scientific) using the Boltzmann equation to determine the inflection point of the transition curve. 

### 4.9. NanoBRET Assay

The assay was performed as previously described [96,97]. In brief, full-length wild-type AURKA, AURKB, and AURKC were obtained as plasmids cloned in frame with a C-terminal NanoLuc-fusion (Promega, Walldorf, Germany; #NV1041, #NV1051, #NV1061, respectively). Plasmids were transfected into HEK293T cells using FuGENE HD (Promega, #E2312), and proteins were allowed to express for 20 h. Serially diluted inhibitor and NanoBRET Tracer K10 (Promega, #N2840) at the respective Tracer *K*_D,app_ (15 nM, 15 nM, and 40 nM for AURKA/B/C) were pipetted into white 384-well plates (Greiner, Frickenhausen, Germany; #781207) using an Echo 550 acoustic dispenser (Beckman Coulter, Krefeld, Germany; former Labcyte, Sheffield, UK). The corresponding protein-transfected cells were added and reseeded at a density of 2.5 × 10^5^ cells/mL after trypsinization and resuspending in Opti-MEM without phenol red (Life Technologies, Darmstadt, Germany). The system was allowed to equilibrate for 2 h at 37 °C/5% CO_2_ prior to BRET measurements. To measure BRET, Nano-Glo Substrate and Extracellular NanoLuc^®^ Inhibitor (Promega, #N2540) were added according to the manufacturer’s protocol and filtered luminescence was measured on a PHERAstar plate reader (BMG Labtech, Ortenberg, Germany) equipped with a luminescence filter pair (450 nm BP filter (donor) and 610 nm LP filter (acceptor)). Competitive displacement data were then graphed using the GraphPad Prism (Version 9.2.0) software using a normalized three-parameter curve fit with the following equation: Y = 100/(1 + 10^X-LogIC50^).

### 4.10. PKA Activity Assay

PKA activity in MCD4 cell lysates was analyzed using the PepTag^®^ Non-Radioactive Protein Kinase Assay (Promega, Walldorf, Germany) according to the manufacturer’s instructions and as previously described [34,36,83,98]. 

### 4.11. Phosphatase Activity Assay

Phosphatase activity in MCD4 cell lysates was determined by para-Nitrophenylphosphate (pNPP)-based activity assays as previously described [34,36,83,99]. Briefly, cells were lysed in SLB without phosphatase inhibitors. Aliquots of 40 µg protein were pipetted into 96-well plates in triplicates. Colorimetric assay buffer (20 mM Tris-HCl, 5 mM MgCl_2_, 1 mM EGTA, 0.02% β-mercaptoethanol, 0.01% (*w*/*v*) BSA; pH 7.5) containing 10 mM of pNPP substrate (NEB, Frankfurt am Main, Germany; #P0757S) was added to each well and incubated for 30 min at 37 °C. The reaction was stopped by adding NaOH, and absorbance at 405 nm was determined with a Spark^®^ 20M Multimode Reader.

### 4.12. Statistical Analyses

Data were analyzed using the GraphPad Prism (Version 9.2.0) software. Brown–Forsythe and Welch ANOVA tests with Games–Howell correction or One-Way ANOVA tests with Tukey or Dunnett correction were used. Significant differences are indicated as **** *p* < 0.0001, *** *p* < 0.001, ** *p* < 0.01, or * *p* < 0.05. Mean plus standard error of the mean (SEM) were plotted.

## Figures and Tables

**Table 1:** AURKA and AURKB inhibitors used in this study.

Compound Name	Formula	Structure	MW (g/mol)	Target
Aurora-A inhibitor I (TC-S 7010)	C_31_H_31_ClFN_7_O_2_	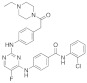	588	AURKA IC_50_ = 3.4 nM [28]
Alisertib (MLN8237)	C_27_H_20_ClFN_4_O_4_	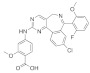	519	AURKA IC_50_ = 1.2 nM [38]
Barasertib-HQPA (AZD1152-HQPA)	C_26_H_30_FN_7_O_3_	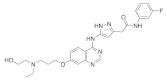	508	AURKB IC_50_ = 0.37 nM [41]

**Table 2:** Novel Aurora-A inhibitor I derivatives.

Compound Name	Formula	Structure	MW (g/mol)	Target ^1^
ERJ2-02 [28]	C_25_H_18_ClFN_5_NaO_3_	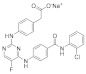	513	AURKA IC_50_ = 1.32 µM AURKB IC_50_ > 50 µM
ERJ2-03	C_29_H_26_ClFN_6_O_3_	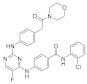	560	AURKA IC_50_ = 1.08 µM AURKB IC_50_ > 50 µM
ERJ2-04	C_31_H_31_ClFN_7_O_3_	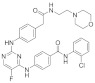	603	AURKA IC_50_ = 554 nM AURKB IC_50_ > 50 µM
ERJ2-05	C_28_H_26_ClFN_6_O_3_	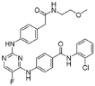	548	AURKA IC_50_ = 239 nM AURKB IC_50_ = 16.7 µM
ERJ2-06	C_25_H_20_ClFN_6_O_2_	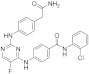	490	AURKA IC_50_ = 165 nM AURKB IC_50_ = 13.1 µM
ERJ2-07	C_27_H_24_ClFN_6_O_2_	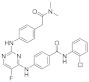	518	Not tested
ERJ2-08	C_27_H_24_ClFN_6_O_3_	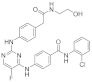	534	AURKA IC_50_ = 237 nM AURKB IC_50_ = 16.1 µM
ERJ2-09	C_18_H_11_FN_4_Na_2_O_4_	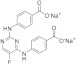	412	AURKA IC_50_ > 50 µM AURKB IC_50_ > 50 µM
ERJ2-10	C_25_H_19_FN_5_NaO_3_	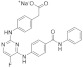	479	AURKA IC_50_ = 22.8 µM AURKB IC_50_ > 50 µM
ERJ2-11	C_31_H_32_FN_7_O_2_	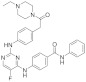	553	AURKA IC_50_ = 767 nM AURKB IC_50_ = 42.9 µM
ERJ2-12	C_25_H_26_FN_6_NaO_3_	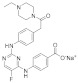	500	AURKA IC_50_ > 50 µM AURKB IC_50_ > 50 µM
ERJ2-13	C_31_H_39_FN_8_O_2_	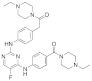	574	AURKA IC_50_ = 37.3 µM AURKB IC_50_ > 50 µM
ERJ2-15	C_31_H_38_FN_7_O_2_	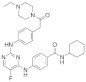	559	Not tested
ERJ2-16	C_31_H_39_FN_8_O_2_	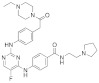	574	AURKA IC_50_ > 50 µM AURKB IC_50_ > 50 µM

^1^ See Figure 6B.

**Figure A1:**
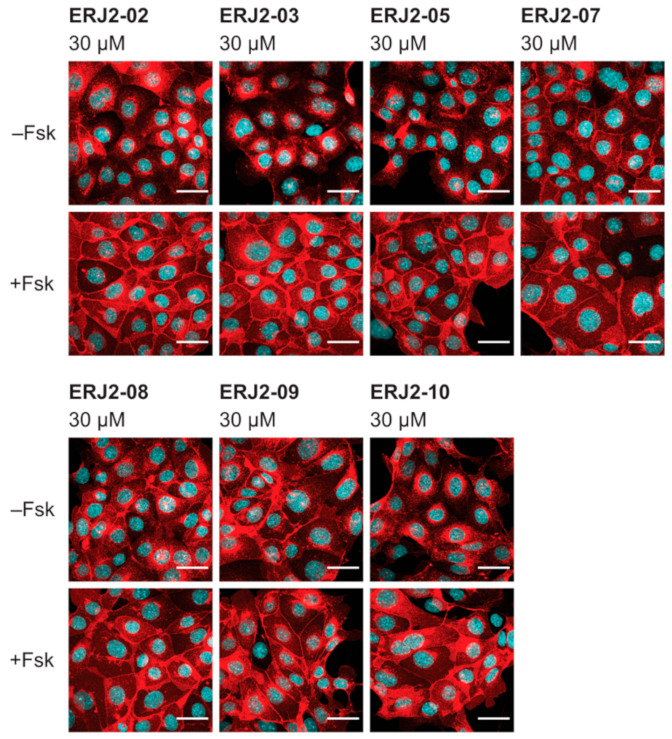
Supplement to Figure 3. Effects of Aurora-A inhibitor I derivatives on the localization of AQP2 in MCD4 cells. MCD4 cells were left untreated or treated with DMSO (0.3%, 1 h) or compound (30 µM, 1 h) alone or in combination with forskolin (Fsk; 30 µM, 30 min) where indicated. AQP2 was detected by immunofluorescence microscopy as described in the legend of Figure 1. Shown are representative images of four independent experiments; scale bar 30 µm.

## Data Availability

The segmentation method for quantification of immunofluorescence images is available at Zenodo (www.zenodo.org), at https://doi.org/10.5281/zenodo.5731600 (last accessed on 8 January 2022).

## Supplementary Materials

The following supporting information can be downloaded at: https://www.mdpi.com/article/10.3390/ijms23020763/s1.
