# Supplementary material for: Aurora Kinase A Is Involved in Controlling the Localization of Aquaporin-2 in Renal Principal Cells
Source: Int J Mol Sci. 2022 Jan 11;23(2):763. doi: 10.3390/ijms23020763 (PMC8776063; doi:10.3390/ijms23020763)
Supplement: Supplementary file 1 [file ijms-23-00763-s001.zip › ijms-1513586-supplementary.pdf]

Supplementary Material

Aurora kinase A is involved in controlling the localization of aquaporin-2 in renal principal cells

Sandrine Baltzer [1,2], Timur Bulatov [2], Christopher Schmied [3], Andreas Krämer [4,5,6], Benedict-Tilman Berger [4,5], Andreas Oder [3], Ryan Walker-Gray [1], Christin Kuschke [1], Kerstin Zühlke [1], Jenny Eichhorst [3], Martin Lehmann [3], Stefan Knapp [4,5,6,7], John Weston [8], Jens Peter von Kries [3], Roderich D. Süssmuth [2] and Enno Klussmann [2,9,*]

1 Max-Delbrück-Center for Molecular Medicine in the Helmholtz Association (MDC), Robert-Rössle-Strasse 10, 13125 Berlin, Germany; sandrine.baltzer@mdc-berlin.de (S.B.)
2 Technische Universität Berlin, Faculty II Mathematics and Natural Sciences, Institute of Chemistry, Chair of Biological Chemistry, Strasse des 17. Juni 135, 10623 Berlin, Germany
3 Leibniz-Forschungsinstitut für Molekulare Pharmakologie (FMP), Robert-Rössle-Strasse 10, 13125 Berlin, Germany
4 Institute of Pharmaceutical Chemistry, Goethe University Frankfurt, Max-von-Laue-Strasse 9, 60438 Frankfurt am Main, Germany
5 Structural Genomics Consortium (SGC), Goethe University Frankfurt, Buchmann Institute for Life Sciences, Max-von-Laue-Strasse 15, 60438 Frankfurt am Main, Germany
6 DKTK (German Translational Research Network), partner site Frankfurt/Mainz, Germany
7 Frankfurt Cancer Institute, 60596 Frankfurt am Main, Germany
8 JQuest Consulting, Carl-Orff-Weg 25, 65779 Kelkheim, Germany
9 DZHK (German Centre for Cardiovascular Research), partner site Berlin, Germany
* Correspondence: enno.klussmann@mdc-berlin.de; Tel.: +49-30-9406-2596; Fax: +49-30-9406-2593

1. General experimental procedures

Commercially available reagents (Fisher Scientific International, Inc., Schwerte, Germany; Carl Roth GmbH and Co. KG, Karlsruhe, Germany; Fluorochem Ltd, Hadfield, UK; Sigma-Aldrich Taufkirchen, Germany; Iris Biotech GmbH, Marktredwitz, Germany; Orpegen, Heidelberg, Germany; ABCR, Karlsruhe, Germany; Alfa Aesar, Karlsruhe, Germany; Merk, Darmstadt; Germany and Acros, Geel, Belgien) and solvents (Fisher Scientific-Acros, Schwerte, Germany) were used without further purification.

Analytical thin layer chromatography was carried out using silica pre-coated aluminum plates from Marcherey-Nagel, Düren, Germany (Alugram® SIL G/UV254, silica gel 60). Analysis was performed by visualizing under UV light (λ = 254 nm) and by staining with $KMnO_4$ solution (3 g $KMnO_4$, 20 g K_2CO_3, 300 ml dist. water, 5 ml NaOH solution (5 %)) or a ninhydrin solution (0.3 g ninhydrin, 2.5 mL glacial acetic acid, 100 mL *n*-BuOH) and subsequent heating.

Flash chromatography was carried out was conducted using silica gel 40-63 µm from VWR Chemicals, Darmstadt, Germany and solvent systems from hexanes (Hex), ethyl acetate (EtOAc), dichloromethane (DCM), chloroform ($CHCl_3$) and methanol (MeOH).

Preparative HPLC was performed with a 1260 Infinity from Agilent Technologies using Sunfire Prep C18 OBD 10 µm, 50x150 mm column (particle size 10 µm x 100 Å) from Agilent Technologies (solvent A: H_2O + 0.1% TFA, solvent B: MeCN + 0.1% TFA, flow rate 50 mL/min) with gradient 10% MeCN to 80% MeCN in 35 min.

^{1}H-NMR spectra were recorded at 298 K using Bruker Avance-II 400 MHz or Bruker Avance-III 500 MHz. TopSpin 3.5 (Bruker, Karlsruhe, Germany) was used for data acquisition and processing. The chemical shifts of synthetic intermediates are reported in parts per million (ppm) using the residual solvent peak as an internal reference (dmso-d_6). Multiplicity (br s = broad singlet, s = singlet, d = doublet, dd = doublet of doublet, t = triplet, q = quartet, m = multiplet).

HPLC-HRMS spectra were recorded on a QTrap LTQ XL (Thermo Fisher Scientific, Waltham, Massachusetts, USA) with an Agilent 1200 Series HPLC-System (Agilent Technologies, Waldbronn, Germany) with a C18 column (50 x 2 mm, particle size 3 µm).

HPLC-ESI-MS coupled measurements were performed on an Orbitrap XL-mass spectrometer from Thermo Scientific(Waltham, MA, USA) coupled with 1200-HPLC from Agilent Technologies using a hypersil 100-C18-column from Thermo Scientific (solvent A: H_2O + 0.1 % HCOOH, solvent B: MeCN+ 0.1% HCOOH; flow rate 1.3 mL/min). Xcalibur (Thermo Scientific) was used for the evaluation of the spectra.

2. Synthesis procedures and compound characterization

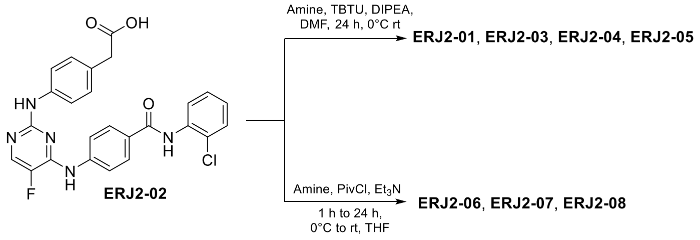

Scheme S1: Synthesis of **ERJ2-01**, **ERJ2-03**, **ERJ2-04**, **ERJ2-05**, **ERJ2-06**, **ERJ2-07**, **ERJ2-08**.

General procedure for the synthesis of compounds ERJ2-01, ERJ2-03, ERJ2-04, ERJ2-05 according to Scheme S1:

To 80 mg (0.16 mmol) of the compound **ERJ2-02**[1] in 3 ml DMF TBTU (62 mg, 0.19 mmol), DIPEA (83 µl, 0.49 mmol) and 0.21 mmol of the corresponding amine (*N*-ethylpiperazine/morpholine/4-morpholineethanamine/2-methoxyethylamine) were added consequently at 0 °C. The reaction mixture was allowed to warm to the room temperature and was stirred for 18 h. The solvent was evaporated at reduced pressure and the residue was purified by preparative HPLC (C18 column).

***N*-(2-chlorophenyl)-4-((2-((4-(2-(4-ethylpiperazin-1-yl)-2-oxoethyl)phenyl)amino)-5-fluoropyrimidin-4-yl)amino)benzamide** (**ERJ2-01**)

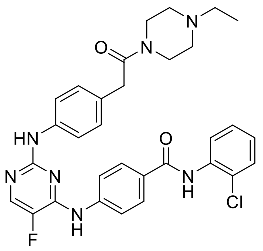

Isolated 36 mg (38%) of the product. **^{1}H-NMR** (500 MHz, dmso-d$_6$): δ (ppm) = 1.19 (t, J = 7.2 Hz, 3 H), 2.92 (m, 2 H), 2.94 (m, 1 H), 3.12 (d, J = 7.5 Hz, 2 H), 3.34 (t, J = 13.0 Hz, 1 H), 3.47 (m, 2 H), 3.73 (d, J = 9.0 Hz, 2 H), 4.21 (d, J = 14.1 Hz, 1 H), 4.46 (d, J = 10.6 Hz, 1 H), 7.14 (d, J = 8.5 Hz, 2 H), 7.31 (td, J = 7.9 Hz, J = 1.6 Hz, 1 H), 7.40 (td, J = 7.9 Hz, J = 1.6 Hz, 1 H), 7.58 (dd, J = 8.1 Hz, J = 1.5 Hz, 1 H), 7.61 (d, J = 8.6 Hz, 2 H), 7.66 (dd, J = 8.1 Hz, J = 1.5 Hz, 1 H), 7.96-8.05 (m, 4 H), 8.21 (d, J = 3.8 Hz, 1 H), 9.42 (s, 1 H), 9.69 (br s, 1 H), 9.76 (s, 1 H), 9.92 (s, 1 H). **HRMS (ESI)** m/z calc for $C_{31}H_{31}ClFN_7O_2$ $[M+H]^+$ 588.2290 found 588.2288.

***N*-(2-chlorophenyl)-4-((5-fluoro-2-((4-(2-morpholino-2-oxoethyl)phenyl)amino)pyrimidin-4-yl)amino)benzamide** (**ERJ2-03**)

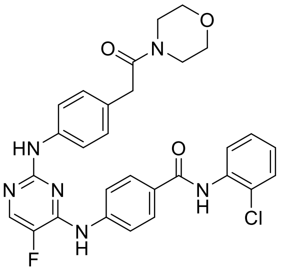

Isolated 40 mg (44%) of the product. **^{1}H-NMR** (500 MHz, dmso-d$_6$): δ (ppm) = 3.44 (m, 2 H), 3.48 (m, 6 H), 3.67 (s, 2 H), 7.15 (d, J = 8.6 Hz, 2 H), 7.30 (td, J = 7.6 Hz, J = 1.5 Hz, 1 H), 7.40 (td, J = 7.6 Hz, J = 1.5 Hz, 1 H), 7.58 (m, 3 H), 7.66 (dd, J = 8.0 Hz, J = 1.5 Hz, 1 H), 7.96-8.05 (m, 4 H), 8.21 (d, J = 3.8

Hz, 1 H), 9.42 (s, 1 H), 9.50 (s, 1 H), 9.87 (s, 1 H), 9.90 (s, 1 H). **HRMS (ESI)** m/z calc for $C_{29}H_{26}ClFN_6O_3$ $[M+H]^+$ 561.1817 found 561.1815.

***N*-(2-chlorophenyl)-4-((5-fluoro-2-((4-(2-((2-morpholinoethyl)amino)-2-oxoethyl)phenyl)amino)pyrimidin-4-yl)amino)benzamide** (**ERJ2-04**)

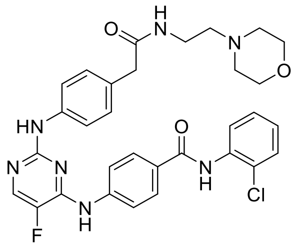

Isolated 40 mg (41%) of the product. **^{1}H-NMR** (500 MHz, dmso-d$_6$): δ (ppm) = 3.07 (m, 2 H), 3.18 (t, , J = 6.3 Hz, 2 H), 3.42 (m, 6 H), 3.62 (m, 2 H), 3.93 (m, 2 H), 7.17 (d, J = 8.6 Hz, 2 H), 7.30 (td, J = 7.6 Hz, J = 1.5 Hz, 1 H), 7.40 (td, J = 7.6 Hz, J = 1.5 Hz, 1 H), 7.57 (dd, J = 8.1 Hz, J = 1.5 Hz, 1 H), 7.60 (d, J = 8.4 Hz, 2 H), 7.65 (dd, J = 8.1 Hz, J = 1.5 Hz, 1 H), 7.96-8.05 (m, 4 H), 8.20 (d, J = 3.7 Hz, 1 H), 8.28 (t, J = 5.6 Hz, 1 H), 9.39 (s, 1 H), 9.47 (s, 1 H), 9.80 (br s, 1 H), 9.93 (s, 1 H). **HRMS (ESI)** m/z calc for $C_{31}H_{31}ClFN_7O_3$ $[M+H]^+$ 604.2239 found 604.2235.

***N*-(2-chlorophenyl)-4-((5-fluoro-2-((4-(2-((2-methoxyethyl)amino)-2-oxoethyl)phenyl)amino)pyrimidin-4-yl)amino)benzamide** (**ERJ2-05**)

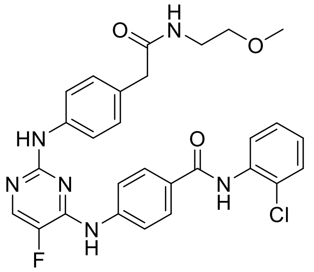

Isolated 35 mg (39%) of the product. **^{1}H-NMR** (500 MHz, dmso-d$_6$): δ (ppm) = 3.16-3.21 (m, 2 H), 3.21 (s, 3 H), 3.30 (t, J = 5.5 Hz, 2 H), 3.38 (s, 2 H), 7.18 (d, J = 8.6 Hz, 2 H), 7.30 (td, J = 7.6 Hz, J = 1.5 Hz, 1 H), 7.40 (td, J = 7.6 Hz, J = 1.5 Hz, 1 H), 7.55 (d, J = 8.4 Hz, 2 H), 7.57 (dd, J = 8.1 Hz, J = 1.5 Hz, 1 H), 7.65 (dd, J = 8.1 Hz, J = 1.5 Hz, 1 H), 7.96-8.04 (m, 4 H), 8.08 (t, J = 5.6 Hz, 1 H), 8.22 (d, J = 4.0 Hz, 1 H), 9.49 (s, 1 H), 9.90 (s, 1 H), 9.92 (s, 1H). **HRMS (ESI)** m/z calc for $C_{28}H_{26}ClFN_6O_3$ $[M+H]^+$ 549.1817 found 549.1815.

General procedure for the synthesis of compounds ERJ2-06, ERJ2-07, ERJ2-08:

To 0.16 mmol of the compound **ERJ2-02**[1] and 0.33 mmol (45 µl) Et_3N in 1 ml THF pivaloyl chloride (40 µl, 0.33 mmol) in 0.5 ml THF was added dropwise at 0 °C. After 5 min at this temperature the mixture was then chilled to -10 °C and 0.48 mmol of the corresponding amine (NH_3 in MeOH/dimethylamine hydrochloride/aminoethanol) was added. The reaction was stirred for 1 h at 0 °C and left overnight at the room temperature. The solvent was evaporated at reduced pressure and the residue was purified by preparative HPLC (C18 column).

4-((2-((4-(2-amino-2-oxoethyl)phenyl)amino)-5-fluoropyrimidin-4-yl)amino)-*N*-(2-chlorophenyl)benzamide (**ERJ2-06**)

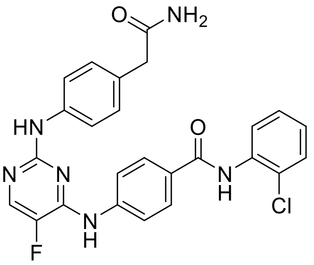

Isolated 20 mg (25%) of the product. **^{1}H-NMR** (500 MHz, dmso-d$_6$): δ (ppm) = 3.36 (s, 2 H), 6.81 (s, 1 H), 7.19 (d, J = 8.6 Hz, 2 H), 7.30 (td, J = 7.7 Hz, J = 1.5 Hz, 1 H), 7.40 (td, J = 7.7 Hz, J = 1.5 Hz, 1 H), 7.57 (d, J = 8.4 Hz, 2 H), 7.66 (dd, J = 8.1 Hz, J = 1.5 Hz, 1 H), 7.96-8.05 (m, 4 H), 8.21 (d, J = 3.7 Hz, 1 H), 9.45 (s, 1 H), 9.84 (s, 1 H), 9.93 (s, 1 H). **HRMS (ESI)** m/z calc for $C_{25}H_{20}ClFN_6O_2$ $[M+H]^+$ 491.1399 found 491.1391.

***N*-(2-chlorophenyl)-4-((2-((4-(2-(dimethylamino)-2-oxoethyl)phenyl)amino)-5-fluoropyrimidin-4-yl)amino)benzamide** (**ERJ2-07**)

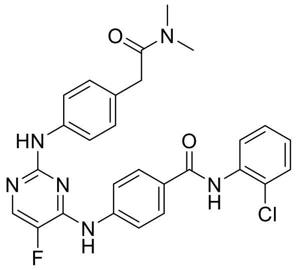

Isolated 25 mg (30%) of the product. **^{1}H-NMR** (500 MHz, dmso-d$_6$): δ (ppm) = 2.81 (s, 3 H), 3.00 (s, 3 H), 3.50 (s, 2 H), 7.17 (d, J = 8.6 Hz, 2 H), 7.30 (td, J = 7.7 Hz, J = 1.5 Hz, 1 H), 7.40 (td, J = 7.7 Hz, J = 1.5 Hz, 1 H), 7.54-7.59 (m, 2 H), 7.65 (dd, J = 8.1 Hz, J = 1.5 Hz, 1 H), 7.96-8.05 (m, 4 H), 8.09 (t, J = 5.6 Hz, 1 H), 8.20 (d, J = 3.7 Hz, 1 H), 9.37 (s,1 H), 9.76 (s, 1 H), 9.90 (s, 1 H). **HRMS (ESI)** m/z calc for $C_{27}H_{24}ClFN_6O_2$ $[M+H]^+$ 519.1712 found 519.1705.

***N*-(2-chlorophenyl)-4-((5-fluoro-2-((4-(2-((2-hydroxyethyl)amino)-2-oxoethyl)phenyl)amino)pyrimidin-4-yl)amino)benzamide** (**ERJ2-08**)

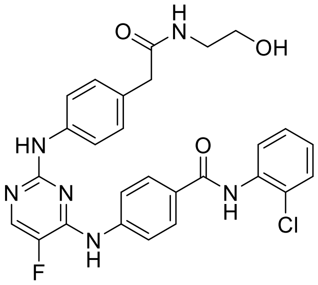

Isolated 27 mg (31%) of the product. **^{1}H-NMR** (500 MHz, dmso-d$_6$): δ (ppm) = 3.10 (q, J = 5.8 Hz, 2 H), 3.37 (q, J = 5.8 Hz, 2 H), 3.38 (s, 2 H), 7.19 (d, J = 8.6 Hz, 2 H), 7.30 (td, J = 7.7 Hz, J = 1.5 Hz, 1 H), 7.40 (td, J = 7.7 Hz, J = 1.5 Hz, 1 H), 7.56 (d, J = 8.4 Hz, 2 H), 7.57 (dd, J = 8.1 Hz, J = 1.5 Hz, 1 H), 7.65 (dd, J = 8.1 Hz, J = 1.5 Hz, 1 H), 7.98 (m, 4 H), 8.21 (d, J = 4.0 Hz, 1 H), 9.46 (s, 1H), 9.86 (s, 1 H), 9.92 (s, 1 H). **HRMS (ESI)** m/z calc for $C_{27}H_{24}ClFN_6O_3$ $[M+H]^+$ 535.1661 found 535.1653.

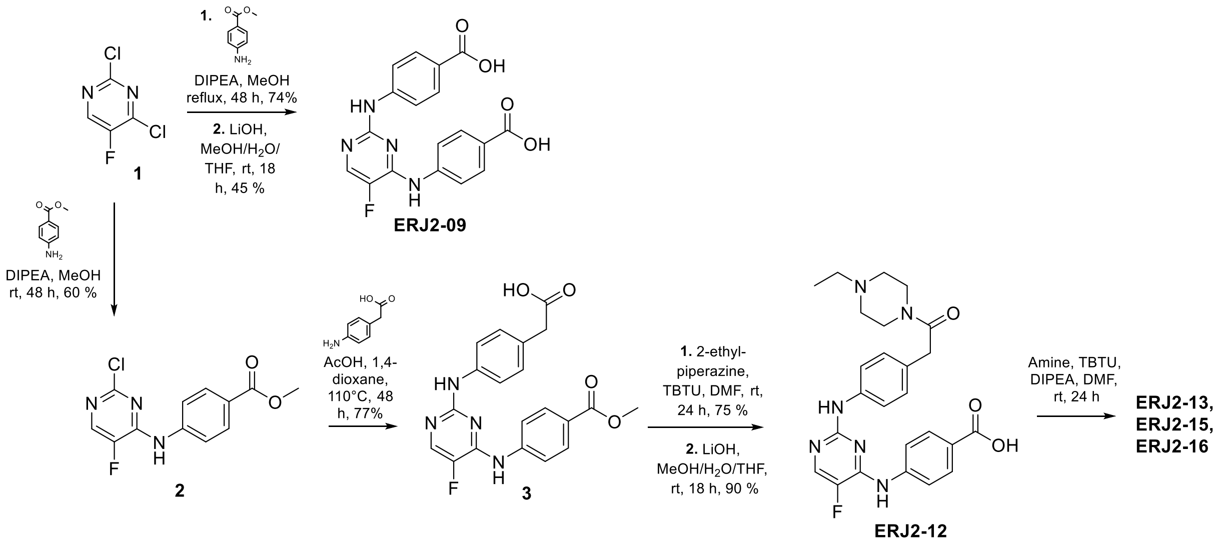

Scheme S2: Synthesis of **ERJ2-09**, **ERJ2-12**, **ERJ2-13**, **ERJ2-15**, **ERJ2-16**.

Synthesis of compounds ERJ2-09, ERJ2-12, ERJ2-13, ERJ2-15, ERJ2-16 according to Scheme S2:

4,4'-((5-fluoropyrimidine-2,4-diyl)bis(azanediyl))dibenzoic acid (**ERJ2-09**)

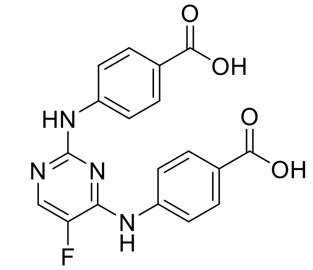

The mixture of 2,4-dichloro-5-fluoropyrimidine **1** (2 g, 12.1 mmol), methyl 4-aminobenzoate (0.61 g, 4 mmol) and 2 ml DIPEA (2 ml, 11.8 mmol) in 20 ml MeOH was heated at reflux for 48 hours. After cooling down a formed product was filtered as a precipitated solid and washed two times with cold methanol to obtain 590 mg (74%) of dimethyl 4,4'-((5-fluoropyrimidine-2,4-diyl)bis(azanediyl))dibenzoate.

To 150 mg (0.38 mmol) of the product in 1 ml MeOH, 1 ml of THF and 1 ml of H_2O was added 91 mg (3.8 mmol) of LiOH and the resulting mixture was stirred for 18 h at the room temperature. After the full conversion of the starting material the solution was acidified with 3 M HCl to pH=3, volatiles were removed under low pressure and the residue was taken up in EtOAc and washed with water (3x). The organic phase was evaporated at reduced pressure and the residue was purified by preparative HPLC (C18 column) to isolate 63 mg (45%) of the product.

^{1}H-NMR (500 MHz, dmso-d_6): δ (ppm) = 7.73-7.77 (m, 4 H), 7.85 (d, J = 8.9 Hz, 2 H), 7.92 (d, J = 8.9 Hz, 2 H), 8.20 (d, J = 3.5 Hz, 1 H), 9.68 (s, 1 H), 9.73 (s, 1 H). **HRMS (ESI)** m/z calc for $C_{18}H_{13}FN_4O_4$ $[M+Na]^+$ 391.0819 found 391.0811.

Methyl 4-((2-chloro-5-fluoropyrimidin-4-yl)amino)benzoate (**2**)

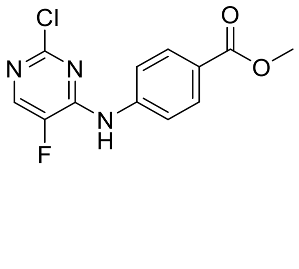

The mixture of 2,4-dichloro-5-fluoropyrimidine **1** (2 g, 12.1 mmol), methyl 4-aminobenzoate (0.61 g, 4 mmol) and 2 ml DIPEA (0.7 ml, 4 mmol) in 20 ml MeOH was stirred at the room temperature for 48 hours. After full conversion of the starting material the solvent was evaporated and the residue was purified by column chromatography to obtain 660 mg (60%) of the monosubstituted product **2**.

^{1}H-NMR (400 MHz, dmso-d_6): δ (ppm) = 3.84 (s, 3 H), 7.89 (d, J = 8.9 Hz, 2 H), 7.97 (d, J = 8.9 Hz, 2 H), 8.42 (d, J = 3.4 Hz, 1 H), 10.28 (s, 1 H).

2-(4-((5-fluoro-4-((4-(methoxycarbonyl)phenyl)amino)pyrimidin-2-yl)amino)phenyl)acetic acid (**3**)

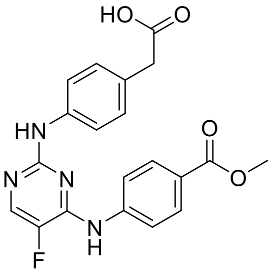

The mixture of 600 mg (2.14 mmol) of methyl 4-((2-chloro-5-fluoropyrimidin-4-yl)amino)benzoate (**2**) and 485 mg (1.43 mmol) of 4-aminophenylacetic acid in 1.8 ml of acetic acid and 7 ml of 1,4-dioxane was heated to 110 °C and stirred for 48 hours at this temperature. After cooling down 5 ml of water was added to the reaction mixture. The formed solid was collected by filtration, washed several times with water and was dried to obtain 655 mg (77%) of the product **3**.

^{1}H-NMR (500 MHz, dmso-d_6): δ (ppm) = 3.57 (s, 2 H), 3.86 (s, 3 H), 7.17 (d, J = 8.3 Hz, 2 H), 7.58 (d, J = 8.3 Hz, 2 H), 7.92 (d, J = 8.9 Hz, 2 H), 8.01 (d, J = 8.9 Hz, 2 H), 8.23 (d, J = 3.8 Hz, 1 H), 9.48 (s, 1 H), 9.89 (s, 1 H).

4-((2-((4-(2-(4-ethylpiperazin-1-yl)-2-oxoethyl)phenyl)amino)-5-fluoropyrimidin-4-yl)amino)benzoic acid (**ERJ2-12**)

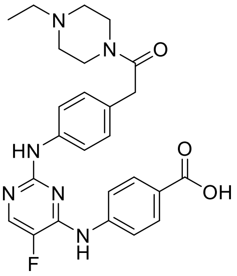

To 230 mg (0.47 mmol) of **3** in 1.5 ml MeOH, 1 ml of THF and 1 ml of H_2O was added 45 mg (1.88 mmol) of LiOH and the resulting mixture was stirred for 18 h at the room temperature. After the full conversion of the starting material the solution was acidified with 3 M HCl to pH=3, volatiles were removed under low pressure and the residue was taken up in EtOAc and washed with water (3x). The organic phase was dried over $MgSO_4$, evaporated at reduced pressure to obtain 200 mg (90%) of the product **ERJ2-12**.

^{1}H-NMR (500 MHz, dmso-d_6): δ (ppm) = 1.21 (t, J = 7.5 Hz, 3 H), 2.86-3.16 (m, 4 H), 3.18-3.63 (m, 6 H), 3.71 (s, 2 H), 7.11 (d, J = 8.3 Hz, 2 H), 7.62 (d, J = 8.3 Hz, 2 H), 7.89 (d, J = 8.9 Hz, 2 H), 8.01 (d, J = 8.9 Hz, 2 H), 8.19 (d, J = 3.8 Hz, 1 H), 9.30 (s, 1 H), 9.68 (s, 1 H). **HRMS (ESI)** m/z calc for $C_{25}H_{27}FN_6O_3$ $[M+H]^+$ 479.2207 found 479.2201.

General procedure for the compounds ERJ2-13, ERJ2-15, ERJ2-16 according to Scheme S2:

To 70 mg (0.15 mmol) of the compound **ERJ2-12** in 2 ml DMF TBTU (58 mg, 0.18 mmol), DIPEA (0.1 ml, 0.60 mmol) and 0.23 mmol of the corresponding amine (*N*-ethylpiperazine/cyclohexylamine/1-pyrrolidineethanamine) was added consequently at 0 °C. The reaction mixture was allowed to warm to

the room temperature and was stirred for 24 hours. The solvent was evaporated at reduced pressure and the residue was purified by preparative HPLC (C18 column).

1-(4-ethylpiperazin-1-yl)-2-(4-((4-((4-(4-ethylpiperazine-1-carbonyl)phenyl)amino)-5-fluoropyrimidin-2-yl)amino)phenyl)ethan-1-one (**ERJ2-13**)

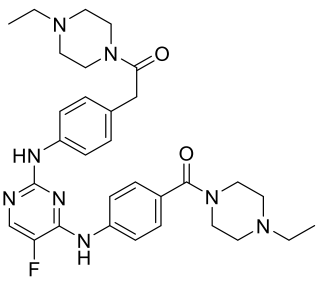

Isolated 28 mg (33%) of the product. **^{1}H-NMR** (400 MHz, dmso-d_6): δ (ppm) = 1.20-1.29 (m, 6 H), 2.57 (s, 2 H), 2.85-3.02 (m, 2 H), 3.04-3.24 (m, 5 H), 3.25-3.43 (m, 2 H), 3.51-3.64 (m, 4 H), 3.70-3.77 (m, 2 H), 4.12-4.35 (m, 3 H), 7.14 (d, J = 8.4 Hz, 2 H), 7.59 (d, J = 8.3 Hz, 2 H), 7.81 (d, J = 8.7 Hz, 2 H), 7.91 (d, J = 8.7 Hz, 2 H), 8.10 (d, J = 8.3 Hz, 1 H), 8.20 (d, J = 3.7 Hz, 1 H), 9.48 (s, 1 H), 9.73 (s, 1 H), 9.81 (br s, 1 H). **HRMS (ESI)** m/z calc for $C_{31}H_{39}FN_8O_2$ $[M+H]^+$ 575.3258 found 575.3243.

***N*-cyclohexyl-4-((2-((4-(2-(4-ethylpiperazin-1-yl)-2-oxoethyl)phenyl)amino)-5-fluoropyrimidin-4-yl)amino)benzamide** (**ERJ2-15**)

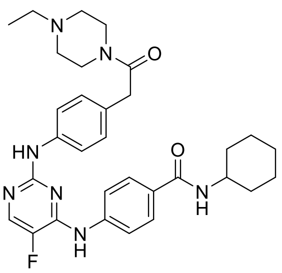

Isolated 19 mg (23%) of the product. **^{1}H-NMR** (500 MHz, dmso-d_6): δ (ppm) = 1.22 (t, J = 7.4 Hz, 3 H), 1.26-1.38 (m, 5 H), 1.59-1.88 (m, 6 H), 2.86-3.00 (m, 4 H), 3.10-3.20 (m, 2 H), 3.28-3.82 (m, 6 H), 7.12 (d, J = 8.3 Hz, 2 H), 7.60 (d, J = 8.3 Hz, 2 H), 7.83 (d, J = 8.7 Hz, 2 H), 7.91 (d, J = 8.7 Hz, 2 H), 8.10 (d, J = 8.3 Hz, 1 H), 8.19 (d, J = 3.7 Hz, 1 H), 9.42 (s, 1 H), 9.68 (s, 1 H), 9.77 (br s, 1 H). **HRMS (ESI)** m/z calc for $C_{31}H_{38}FN_7O_2$ $[M+H]^+$ 560.3149 found 560.3142.

4-((2-((4-(2-(4-ethylpiperazin-1-yl)-2-oxoethyl)phenyl)amino)-5-fluoropyrimidin-4-yl)amino)-N-(2-(pyrrolidin-1-yl)ethyl)benzamide (**ERJ2-16**)

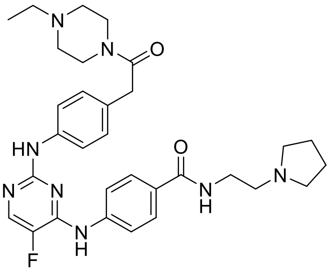

Isolated 15 mg (18%) of the product. **^{1}H-NMR** (500 MHz, dmso-d_6): δ (ppm) = 1.21 (t, J = 7.1 Hz, 3 H), 1.75-2.13 (m, 4 H), 2.79-4.36 (m, 20 H), 7.13 (d, J = 8.3 Hz, 2 H), 7.59 (d, J = 8.3 Hz, 2 H), 7.81 (d, J = 8.7 Hz, 2 H), 7.90 (d, J = 8.7 Hz, 2 H), 8.10 (d, J = 8.3 Hz, 1 H), 8.18 (d, J = 3.7 Hz, 1 H), 9.35 (s, 1 H), 9.65 (s, 1 H), 9.68 (br s, 1 H). **HRMS (ESI)** m/z calc for $C_{31}H_{39}FN_8O_2$ $[M+H]^+$ 575.3258 found 575.3247.

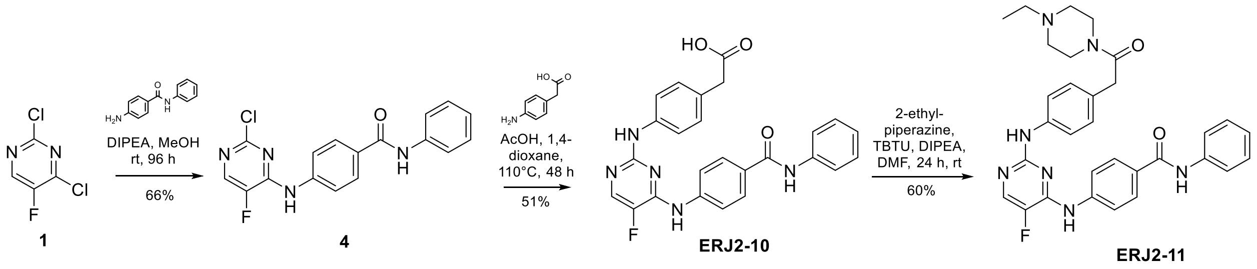

Scheme S3: Synthesis of **ERJ2-10**, **ERJ2-11**.

Synthesis of compounds ERJ2-10, ERJ2-11 according to Scheme S3:

4-((2-chloro-5-fluoropyrimidin-4-yl)amino)-N-phenylbenzamide

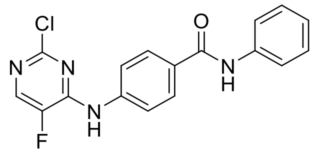

The mixture of 2,4-dichloro-5-fluoropyrimidine **1** (1 g, 6.0 mmol), 4-amino-*N*-phenylbenzamide (0.5 g, 2.3 mmol) and 0.5 ml DIPEA (2.9 mmol) in 20 ml MeOH was stirred at the room temperature for 96 hours. After full conversion of the starting material the precipitate was collected by filtration and washed with cold MeOH to obtain 533 mg (66%) of the monosubstituted product **4**.

^{1}H-NMR (500 MHz, dmso-d_6): δ (ppm) = 7.10 (t, J = 7.3 Hz, 1 H), 7.35 (t, J = 7.9 Hz, 2 H), 7.78 (d, J = 8.5 Hz, 2 H), 7.87 (d, J = 8.7 Hz, 2 H), 7.99 (d, J = 8.7 Hz, 1 H), 8.40 (d, J = 3.4 Hz, 2 H), 10.18 (s, 1 H), 10.26 (s, 1 H).

2-(4-((5-fluoro-4-((4-(phenylcarbamoyl)phenyl)amino)pyrimidin-2-yl)amino)phenyl)acetic acid (ERJ2-10)

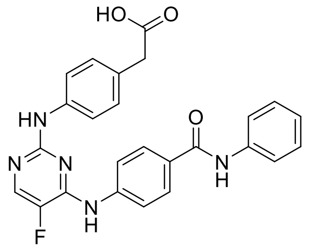

The mixture of 200 mg (0.58 mmol) of 4-((2-chloro-5-fluoropyrimidin-4-yl)amino)-*N*-phenylbenzamide (**4**) and 162 mg (1.07 mmol) of 4-aminophenylacetic acid in 0.6 ml of acetic acid and 2 ml of 1,4-dioxane was heated to 110 °C and stirred for 48 hours at this temperature. After cooling down 2 ml of water was added to the reaction mixture. The formed solid was collected by filtration, washed several times with water and was dried to obtain 136 mg (51%) of the product **ERJ2-10**.

^{1}H-NMR (500 MHz, dmso-d_6): δ (ppm) = 3.53 (s, 2 H), 7.10 (tt, J = 7.4 Hz, J = 0.8 Hz, 1 H), 7.19 (d, J = 8.6 Hz, 2 H), 7.36 (t, J = 8.2 Hz, 2 H),7.59 (d, J = 8.4 Hz, 2 H), 7.80 (d, J = 8.6 Hz, 2 H), 7.94-8.04 (m, 4 H), 8.22 (d, J = 4.0 Hz, 1 H), 9.53 (s, 1 H), 9.87 (s, 1 H), 10.14 (s, 1 H). **HRMS (ESI)** m/z calc for $C_{25}H_{20}FN_5O_3$ $[M+H]^+$ 458.1628 found 458.1620.

4-((2-((4-(2-(4-ethylpiperazin-1-yl)-2-oxoethyl)phenyl)amino)-5-fluoropyrimidin-4-yl)amino)-*N*-phenylbenzamide (ERJ2-11)

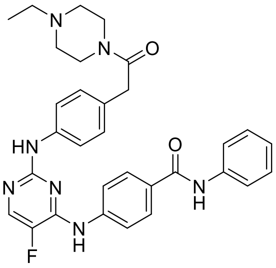

To 60 mg (0.13 mmol) of the compound **ERJ2-10** in 2 ml DMF TBTU (50 mg, 0.16 mmol), DIPEA (0.1 ml, 0.60 mmol) and 25 µl (0.20 mmol) of *N*-ethylpiperazine was added consequently at 0 °C. The reaction mixture was allowed to warm to the room temperature and was stirred for 24 hours. The solvent was evaporated at reduced pressure and the residue was purified by preparative HPLC (C18 column) to obtain 43 mg (60%) of the product **ERJ2-11**.

^{1}H-NMR (500 MHz, dmso-d$_6$): δ (ppm) = 1.21 (t, J = 7.2 Hz, 3 H), 2.85-2.97 (m, 3 H), 3.10-3.18 (m, 2 H), 3.41-3.52 (m, 2 H), 3.62-3.97 (m, 3 H), 4.21 (d, J = 13.9 Hz, 1 H), 4.47 (d, J = 10.8 Hz, 1 H), 7.08-7.16 (m, 3 H), 7.36 (t, J = 8.4 Hz, 2 H), 7.61 (d, J = 8.5 Hz, 2 H), 7.79 (d, J = 8.5 Hz, 2 H), 7.93-8.04 (m, 4 H), 8.20 (d, J = 3.7 Hz, 1 H), 9.42 (s, 1 H), 9.66 (br s, 1 H), 9.47 (s, 1 H), 10.15 (s, 1 H). (**HRMS (ESI)** m/z calc for $C_{31}H_{32}FN_7O_2$ $[M+H]^+$ 554.2680 found 554.2670.

3. References

1. Aliagas-Martin, Ignacio, et al. A class of 2, 4-bisanilinopyrimidine Aurora A inhibitors with unusually high selectivity against Aurora B. *Journal of Medicinal Chemistry*, 52 (10), **2009**, 3300-3307.
